# Potential of digital applications for self-management and other outcomes in inflammatory rheumatic diseases: a systematic literature review

**DOI:** 10.3389/fmed.2025.1617151

**Published:** 2025-07-09

**Authors:** Alina Volkmar, Cay-Benedict von der Decken, Stefan Kleinert, Kirsten Karberg, Georg Gauler, Michael Klennert, Jana Klennert, Peer Malte Aries, Sonja Froschauer, Sarah Wildenhain, Theresia Muth, Susanna Späthling-Mestekemper, Christoph Kuhn, Wolfgang Vorbrüggen, Martin Welcker, Peter Bartz-Bazzanella, Matthias Englbrecht

**Affiliations:** ^1^Faculty of Life Sciences, University of Applied Sciences, Hamburg, Germany; ^2^RHADAR – RheumaDatenRheport GbR, Erlangen, Germany; ^3^Medizinisches Versorgungszentrum, Rheumatologie, Stolberg, Germany; ^4^Verein zur Förderung der Rheumatologie e.V., Würselen, Germany; ^5^Klinik für Internistische Rheumatologie, Rhein-Maas-Klinikum, Würselen, Germany; ^6^Praxisgemeinschaft Rheumatologie-Nephrologie, Erlangen, Germany; ^7^Rheumatologisches Versorgungszentrum Steglitz, Berlin, Germany; ^8^Rheumatology Practice, Osnabrück, Germany; ^9^STAR Healthcare Management GmbH, Köln, Germany; ^10^Immunologikum Hamburg, Hamburg, Germany; ^11^BDRh Service GmbH, Grünwald, Germany; ^12^Rheumapraxis München, München, Germany; ^13^Praxis für Rheumatologie, Karlsruhe, Germany; ^14^M.B.W.-Welcker GbR, Planegg, Germany; ^15^Freelance Healthcare Data Scientist, Greven, Germany

**Keywords:** digital applications, inflammatory rheumatic diseases, systematic review, self-management, pain, depression, self-care, functional impairment

## Abstract

**Introduction:**

Inflammatory rheumatic diseases (IRDs) are chronic autoimmune conditions affecting the musculoskeletal system, leading to pain, disability, and reduced quality of life. Despite advances in medical treatments, barriers such as delayed diagnosis, workforce shortages, and low adherence to self-management strategies remain critical challenges. Digital health applications are emerging as promising tools to enhance disease management. The aim of this study was to conduct a systematic literature review (SLR) to evaluate self-care outcomes associated with digital health applications in IRDs.

**Methods:**

We conducted a systematic literature review according to PRISMA guidelines across four electronic databases (PubMed, Embase, CINAHL, Cochrane) from inception to July 2024. Randomized controlled trials (RCTs) and systematic reviews focusing on digital interventions to promote self-management and self-care in individuals with IRDs were included. Key outcomes were extracted and the quality of the included studies was assessed using the Critical Appraisal Skills Programme (CASP) checklist.

**Results:**

Fifteen RCTs with a total of 1912 participants were analyzed. Primary outcomes, including self-management/self-care, showed mixed results. Some studies demonstrated significant improvements in self-efficacy, pain reduction, depression/anxiety symptoms, and physical function, while others showed no notable changes. Secondary outcomes, including disease activity and medication adherence, revealed heterogeneous findings. Variability in study design, sample size, and intervention duration posed challenges for drawing definitive conclusions about the effectiveness of digital interventions.

**Discussion:**

Digital health applications show promise as cost-effective and scalable solutions to complement standard IRD care by empowering patients in their disease management. However, significant heterogeneity and limited generalizability highlight the need for more robust and long-term research to validate the efficacy of specific tools and identify best options for improving outcomes. Tailored digital interventions could bridge the gap in care for IRD patients and support their autonomy.

## 1 Introduction

The umbrella term inflammatory rheumatic diseases (IRDs) refers to a group of autoimmune diseases that are highly diverse and often chronic. IRDs are characterized by systemic inflammation that typically affects the joints, tendons, muscles, ligaments and bones, but may also involve internal organs such as the heart, lungs, and kidneys (1, 2). Conditions considered to be IRDs include rheumatoid arthritis (RA), psoriatic arthritis (PsA), axial spondylarthritis (axSpA), systemic lupus erythematosus (SLE), and gout.

IRDs are among the most common chronic diseases worldwide and can affect all age groups. If not properly managed, they can result in irreversible joint damage and cartilage breakdown. The resulting disease symptoms may manifest as considerable pain, disability, and decreased quality of life (QoL) (3). IRDs are further complicated by frequent physical comorbidities, including cardiovascular diseases and osteoporosis (4), and psychological comorbidities, most notably anxiety and depression (5, 6). In addition to the physical and mental effects of IRDs, socioeconomic consequences for individuals, caretakers, and society are substantial, particularly with respect to decreased work productivity and early retirement (7).

In Germany, IRDs affect approximately 2.2 to 3.0% of the adult population, which equates to approximately 1.5–2.1 million individuals (8). Although recent global prevalence data for IRDs are not available, the most common IRD, RA, currently affects an estimated 17.6 million people worldwide; forecasts indicate this number will almost double to 31.7 million by 2050 (9).

The increasing number of people with IRDs is juxtaposed with a shortage of rheumatologists in Germany (10) and elsewhere (11–14). In some developed countries, rheumatology workforces are about half the recommended level (10, 11, 14). These deficits are further exacerbated by the complexity of current rheumatology care, which typically involves “treat to target” strategies (15–17) and close monitoring of potential treatment side effects (14). Shortages in the rheumatology workforce have the potential to jeopardize optimal rheumatologic patient care by delaying early diagnosis and treatment and impacting patient follow-up care (10).

With the rising numbers of patients with IRDs and the current lack of patient care resources, innovative solutions are necessary. Digital healthcare applications (apps) present a promising route for addressing these challenges. Corresponding tools for IRDs range from those designed to streamline the diagnostic process to those that monitor treatment effectiveness and tolerability. Additionally, apps can aid in the identification and treatment of comorbidities, convey patient education and rehabilitation information, and provide tailored expert knowledge for both patients and medical professionals (18, 19). Both clinicians and patients have expressed strong interest in using digital healthcare tools to assist with disease management (18). In 2018, 67% of rheumatologists planned to incorporate medical apps into routine care, up from 47% in 2016 (18).

In Germany, the trend toward digital health solutions has been further propelled by the introduction of the Digital Healthcare Act (DVG), which came into effect in 2020. The DVG includes provisions for patients to have access to specific apps (“prescription apps”), known as digital health applications, which are reimbursed by statutory health insurance providers (18). Other countries are introducing similar programs, with various reimbursement models (20, 21).

An important use of digital health applications is to empower patients to take a proactive role in managing their health by providing guidance on self-management and self-care. In this context, self-management, which encompasses self-care and self-efficacy and is also termed patient activation, refers to the ability and willingness of patients to manage their health conditions (22). There are a multitude of opportunities to integrate apps designed to improve self-management into the care of patients with IRDs. For instance, digital interventions can reduce pain interference in chronic pain conditions, including IRDs (23). Digital strategies may also be able to enhance long-term treatment adherence, which can be suboptimal in patients with IRDs, leading to greater disability and reduced effectiveness of therapy (24). Another possible avenue is management of depression and anxiety (25), which may affect self-management behavior and treatment adherence (6). These findings suggest that digital health applications have the potential to provide a valuable resource for managing the physical and mental health aspects of IRDs.

The goal of this systematic literature review (SLR) was to investigate the current landscape and relevance of digital tools in enhancing self-management and related outcomes in patients with IRDs. This assessment may be of use in future development of digital interventions for the self-management of IRDs.

## 2 Methods

The SLR presented here adhered to the Preferred Reporting Items for Systematic Reviews and Meta-Analyses (PRISMA) guidelines, a 27-item checklist designed to enhance the completeness and transparency of systematic review reporting (26). An electronic databank search was conducted in June and July 2024 across four databases: PubMed, Cochrane, Embase, and Cumulated Index in Nursing and Allied Health Literature (CINAHL). Those four databases were chosen as they collectively offer extensive coverage of medical, clinical, and allied health literature necessary to capture the various dimensions of the topic. Because of the study design, this SLR was not registered in a clinical study database. A separate protocol was not prepared.

Studies were sought that focused on different digital tools such as websites, apps, and wearable devices for self-management and self-care in people with IRDs. The aim was to evaluate whether such digital programs (in addition to usual care) resulted in improvement in patients’ self-management of the disease, including reduction in pain, fewer symptoms of depression and anxiety, and less functional impairment.

### 2.1 Search strategy

The literature search included published articles (including those published online first) listed in the specified databases up until the day of the search (July 10, 2024 for PubMed and July 15, 2024 for Cochrane, Embase, and CINAHL). All included studies were required to be published in German or English. The framework for the search was based on the Patients, Intervention, Comparator, Outcome (PICO) format (27) (Table 1). The search terms included Medical Subject Headings (MeSH) terms, keywords, and wildcard terms found in the title or abstract. Briefly, included studies involved patients with IRDs, interventions with digital tools, a comparison to patients who did not use digital tools, and an outcome involving self-management/self-care and related assessments as shown in Table 1. The abbreviated PubMed search strategy is shown in Table 2 and detailed search strategies for the four databases are provided in Supplementary file S1. Only studies that were either a randomized controlled trial (RCT) or an SLR/meta-analysis were eligible for inclusion.

**Table 1:** Digital application systematic literature review entry criteria.

Criteria	Included studies	Excluded studies
*PICO criteria*		
Population (P)	Adults (≥18) with IRDs	Children or individuals with no IRD
Intervention (I)	Digital tools involving aspects of self-management/self-care	Non-digital tools or digital tools evaluating other aspects of IRD management
Comparison (C)	No use of digital tools (e.g., routine or usual care)	No comparison group
Outcomes (O)	Changes in: *Primary* Self-management/self-care, including physical activity Pain Depression and anxiety Functional impairment *Secondary* Disease activity Work productivity Medication adherence QoL Fatigue	Changes in outcomes not related to the specified outcomes
*Additional criteria*		
Type of study	RCTs^a^	Observational, case studies
Language	Published in English or German	Published in other languages
Date	Any time up to date of search (July 10 or July 15, 2024)	Listed in database after date of search

Only published, randomized studies were included.

^a^Systematic literature reviews/meta-analyses dealing with the relevance of digital tools for adults with IRDs were allowed in the original search, but none of the identified studies were ultimately included in this systematic review.

IRD, inflammatory rheumatic disease; QoL, quality of life; RCT, randomized clinical trial.

**Table 2:** Abbreviated PubMed search strategy.

Sequence	Search term	Retrieved sequential hits
#1	(arthritis, rheumatoid[mesh] OR rheumatoid arthritis[text word] OR chronic polyarthritis[text word] OR ((rheumatoid[text word] OR reumatoid[text word] OR rheumatic[text word] OR reumatic[text word] OR rheumat*[text word] OR reumat*[text word]) AND (arthrit*[text word] OR artrit*[text word] OR diseas*[text word] OR condition*[text word] OR nodule*[text word])) OR Arthritis, Psoriatic[mesh] OR (psoria*[text word] AND (arthriti*[text word] or arthropath*[text word])) OR systemic lupus erythematosus[text word] OR lupus erythematosus, systemic[mesh] OR Lupus[text word] OR lupus nephritis[mesh] OR (bechterew*[text word] AND disease[text word]) OR Spondylarthropathies[mesh] OR (ankylos*[text word] OR spondyl*[text word] OR axial spondyl*[text word]) OR (bekhterev*[text word] OR bechterew*[text word]) OR (Marie[text word] AND struempell*[text word]) OR Bechterew’s disease[text word] OR undifferentiated arthritis[text word])	394,715
#2	(appl*[title] OR online*[title] OR web-bas*[title] OR mobile[title] OR digital[title] OR program*[title] OR education[title] OR e-health[title] OR telemedicine[title] OR mHealth[title] OR digital health[title] OR online platform[title] OR internet [title] OR mindfulness*[title] OR relaxation[title] OR stress-reduc*[title] OR breath*[title] OR forest-bathing[title] OR shinrin-yoku[title] OR self-acceptance[title] OR psycho* intervention* [title])	1,073,053
#3	(standard of care[text word] OR gold standard [text word] OR routine*[text word] OR usual care[text word] OR conventional treatment[text word] OR standard therapy[text word] OR placebo [text word] OR clinical practice [title] OR practice [title] OR care [title] OR randomiz*[text word] OR randomis*[text word])	2,605,596
#4	(self-management[text word] OR self-care[text word] OR health maintenance[text word] OR self-empowerment[text word] OR physical function*[text word] OR physical abilities[text word] OR bodily capabilities[text word] OR pain[mesh] OR pain measurement[mesh] OR pain intensity[text word] OR depression[mesh] OR depressive disorder[mesh] OR depressed mood OR depressiv*[text word] OR psychological distress[title] OR impact of disease[text word] OR disease impact[text word] OR illness consequences[text word] OR disease effects[text word] OR quality of life[mesh] OR „health-related quality of life“[text word] OR treatment satisfaction[text word] OR Outcome Assessment, Health Care[mesh] OR Outcome and Process Assessment, Health Care[mesh] OR disease activity[text word] OR patient compliance[mesh] OR adherence [text word])	2,734,184
#5	#1 AND #2 AND #3 AND #4	509

Detailed search strategies for PubMed, Cochrane, Embase, and the Cumulated Index in Nursing and Allied Health Literature (CINAHL) are provided in Supplementary file S1.

### 2.2 Selection of studies

For the purposes of this SLR, we chose four primary outcomes of interest: self-management (including patient activation and physical activity), pain, depression/anxiety, and functional impairment (including impairment in activities of daily living). These outcomes were not necessarily designated as primary outcomes in the original study. We also evaluated several secondary outcomes for this systematic review, including disease activity, work productivity, medication adherence, QoL, including health-related QoL (HR-QoL), and fatigue (Table 1).

The suitability of studies was evaluated by two reviewers (AV and ME) using Rayyan as a digital platform for literature screening (28). Discrepancies were discussed to ensure consistency of inclusion and exclusion criteria. Studies not meeting the inclusion criteria based on the title or abstract were excluded. A reason for exclusion was recorded for each study to document the exclusion process and generate a list of exclusion reasons. The reasons were prioritized and coded as follows:

1. Wrong population (not an IRD).
2. Wrong outcome (not a self-management intervention or lacking self-management components).
3. Wrong intervention (not a digital intervention).
4. Wrong study design (not an RCT or an SLR/meta-analysis).

### 2.3 Data extraction and quality assessment

The selection of information to be extracted from the identified studies and included in the results table was initially made by AV and subsequently cross-checked by ME. After discussing and agreeing on the appropriate table headings, AV extracted the data to complete the results table. The results were manually entered into the table to minimize the risk of errors.

The quality of the studies was assessed using the established Critical Appraisal Skills Programme (CASP) checklist which is a standardized critical appraisal tool for RCTs (29). The CASP checklist is broadly divided into four sections: Section A evaluates the appropriateness of the study design for a RCT, section B assesses the methodological rigor of the study, section C investigates the reported results, and section D considers the applicability of these results in a local context (29).

### 2.4 Statistical analyses

Extracted data, including study characteristics and reported outcomes of interest, were summarized descriptively. If the mean age for the full study cohort was not reported, a weighted mean age was calculated using the mean ages of the intervention and control groups. No additional statistical analyses were conducted. Effect sizes and *p* values included in this SLR are as reported by the cited study.

## 3 Results

### 3.1 Retrieved articles

**Figure 1:**
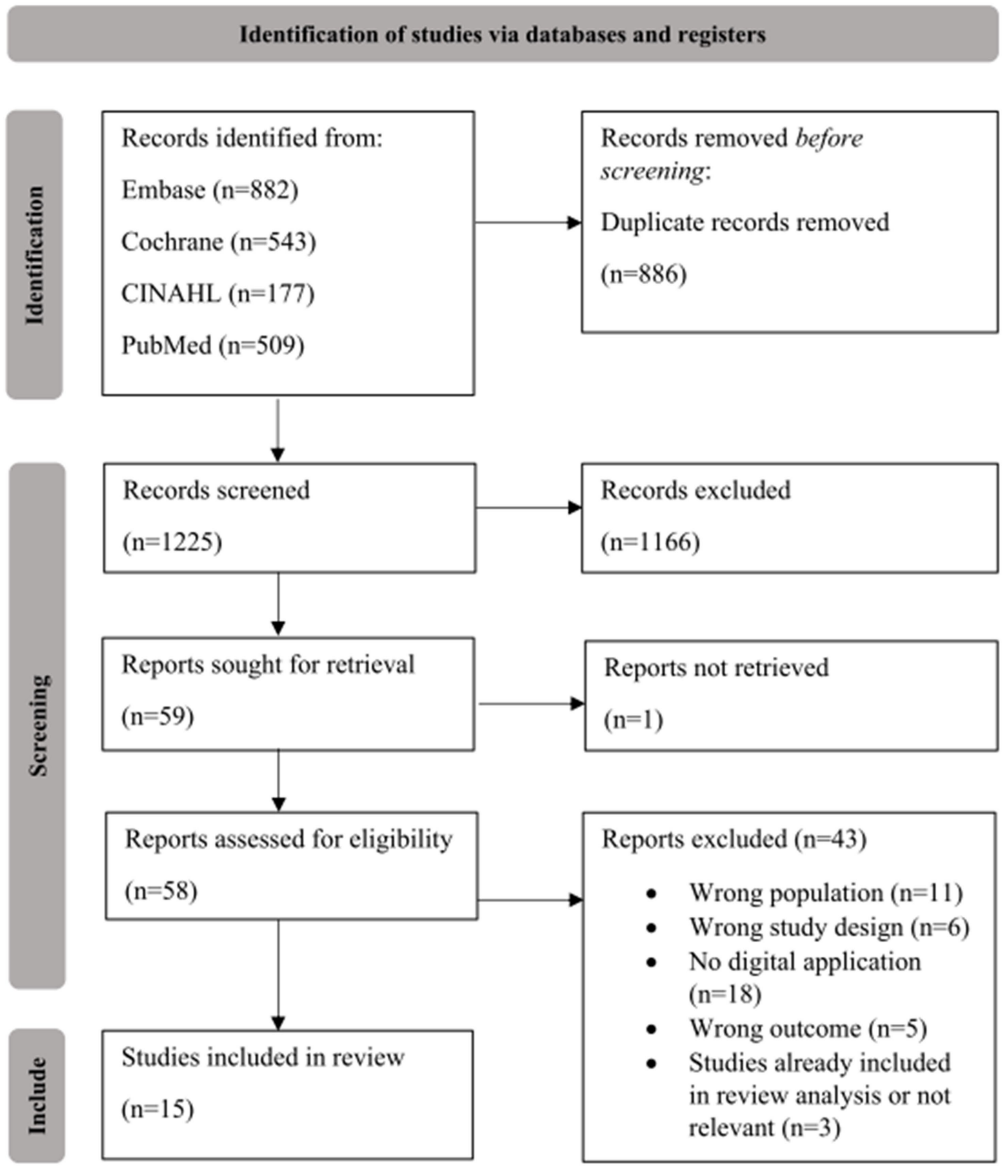
PRISMA flow chart. The suitability of studies was evaluated by two reviewers (AV and ME).

A total of 2111 records were identified and 886 duplicate records were removed, leaving 1225 records for title/abstract screening (Figure 1). Of these, 1166 were excluded, usually due to not evaluating an outcome of interest or not involving adults with IRDs. Fifty-nine reports were retrieved and assessed for eligibility, of which 15 met all study eligibility criteria. The two most common reasons for exclusion during this phase of selection were wrong intervention (no digital application) and wrong population (Figure 1).

Although SLRs and meta-analyses were eligible for study inclusion, all included studies were RCTs. Three SLRs, Srikesavan et al. (30), Griffiths et al. (31), and Ritschl et al. (32), were initially marked as included after the full-text screening. However, during data extraction, these studies were ultimately excluded for the following reasons: Srikesavan et al. (30) included studies already identified as eligible for the current systematic review and Griffiths et al. (31) and Ritschl et al. (32) included studies that did not meet the inclusion criteria for our analysis.

### 3.2 Characteristics of included studies

#### 3.2.1 Study overview

The 15 RCTs included in this SLR encompassed a total of 1912 participants (Table 3) (23, 33–46). Only one of the studies had a sample size over 200, five had a sample size between 150 and 200, six had a sample size between 100 and 150, and three had a sample size <50. Data of the included studies were from Europe (*n* = 8, four from the Netherlands and one each from Spain, Germany, Switzerland and Denmark), North America (*n* = 6, three from Canada and three from the US), and Asia (*n* = 1, from China). Publication dates ranged from 2006 to 2025; the article published in 2025 was published online first on December 28, 2023 (39). The proportion of women across the studies varied widely, with reported values ranging from 21 to 96%, while three studies did not report this information or reported this information for the control and intervention groups separately but not for the overall sample. The mean age of participants spanned from 30 to 62 years (Table 3).

**Table 3:** Overview of the 15 studies included in the systematic review.

First author [publication year] (ref number)	Year(s) of study	Country	IRD	N (% female)	Mean (SD) age in years	Intervention	Control	Duration
Allam [2015] (33)	2013	Switzerland	RA	155 (46%)	58 (12.3)	Website: ONESELF (4 groups with access to different sections/ features): 1. Informational sections only 2. Informational and social support sections 3. Informational and gaming sections 4. Informational, social support, and gaming sections	Standard care, delayed access to ONESELF after study completion	16 wks
Allen [2021] (34)	2018–2019	USA	SLE	60 (95%)	49 (NR)^a^	Internet-based training on pain coping skills (PainTRAINER) 8 weekly modules on pain coping strategies (30–45 min) Guided feedback, interactive exercises, animated demonstrations, automated email reminders	Standard care, delayed intervention after completion of follow-up assessments	6 wks
Ferwerda [2017] (35)	2009–2013	The Netherlands	RA and psychological distress	133 (64%)	56 (10)	Internet-based cognitive behavioral intervention 1 to 4 tailored intervention modules (pain and functional disability, fatigue, negative mood or social functioning) Assignments, psychoeducational texts and cognitive strategies	Standard rheumatology care	9 to 65 wks (mean [SD], 26 [12] wks)
Khan [2020] (36)	2017–2018	USA	SLE and a stable dose of ≥1 rheumatologic drug for ≥3 months	46 (96%)	43 (NR)	Mobile smartphone app Tracker for lifestyle activities, symptoms Software that analyzes and organizes data Web portal that presents data to health coach	Standard rheumatology care	16 wks
Knudsen [2024] (37)	2021–2023	Denmark	RA diagnosis within past 3 months	175 (61%)	59 (NR)	Web-based digital patient education program regarding disease-specific knowledge Mandatory module: typical disease course, causes, symptoms, treatment Optional modules including potential comorbidities, guidance for managing symptoms and coping with RA	1-h session of face-to-face patient education by rheumatology nurse	1 year
Kurt [2024] (38)	2022–2023	Germany	RA, SpA, or PsA	158 (73%)	53 (12)	Mobile app (Mida Rheuma) with individualized lifestyle counselling Patient-reported outcomes Disease burden Healthy mediterranean diet Sports and physical activity Mental health Non-smoking Individualized treatment action plans 7–11 days long	Mida Rheuma app to assess patient-reported outcomes only	12 wks
Li [2025] (39)^b^	2019–2022	Canada	RA	131 (92%)	56 (13)	Web/mobile app OPERAS and fitness tracker (Fitbit) Monitor disease activity, symptoms, medication use, fatigue, depression, sleep quality 6 PT phone counselling sessions One 2-h physical activity session with group and individual counselling	Standard medical treatment and monthly e-newsletter unrelated to RA	26 wks
Li [2020] (40)	2017–2019	Canada	RA or SLE	118 (92%)	56 (13)	Web-based app FitVitz and fitness tracker (Fitbit) with account access In-person session with 20 min group education, 30 min individual counselling with PT Ability to track fitness goals 4 bi-weekly PT phone calls	Standard care, delayed intervention (after 10 wks)	8 wks
Lorig [2008] (41)	2004–2005	Canada	RA	144 (NR^b^)	NR (NR)^c^	Internet-based arthritis self-management program Learning and discussion centers Exercise logs and programs Medication diaries Arthritis helpbook	Standard care and $10 Amazon.com gift card for each questionnaire completed	6 wks
Pouls [2022] (42)	2019–2021	The Netherlands	RA and using DMARDs	221 (73%)	61 (12)	Mobile (smartphone/tablet) game-based intervention: players completed behavioral tasks to unlock games Crossword, sudoku, word search, tangram	Standard care, delayed intervention (after 12 wks)	12 wks
Rodríguez Sánchez-Laulhé [2022] (23)	2020–2021	Spain	RA ≥ 2 years, hand involvement, current pain and siability	30 (61%)	56 (13)	Mobile CareHand app Exercise videos and diary Exercise protocol to be performed 4 times/wk., 15–20 min each Advice on diet and joint protection, self-management Telephone follow-up calls	Standard care with written exercise program and recommenda-tions	12 wks
Shigaki [2013] (43)	2003-2010^d^	USA	RA on stable medication for ≥3 months	106 (93%)	50 (NR)	Website RAHelp Self-management and educational programs Social networking applications (news feature) Assessment and monitoring tools Database for leaders Weekly telephone support for 15–30 min	Standard care, delayed intervention (after 10 wks)	10 wks
Song [2022] (44)^e^	2017	China	AS	118 (21%)	30 (8)	Mobile social networking app “WeChat” Educational information, medication, exercise, daily life management, psychological support, self-assessment 4 individual educational sessions via WeChat	Standard care with health advice via paper handouts and in-person consultations by a nurse	12 wks
van den Berg [2006] (45)	2002–2004	The Netherlands	RA	160 (77%)	50 (NR)^a^	Website “cybertraining.nl” individualized physical activity program Personalized exercises, bicycle ergometer, advice for additional physical activities Quarterly group meetings Individual supervision by PTs	General training (access to part of web site with general exercises, informational material)	1 year
Zuidema [2019] (46)	2013–2014	The Netherlands	RA	157 (NR)	62 (NR)^a^	Web-based self-management program Modules with videos, information, exercises Diary to track symptoms	Standard care	1 year

^a^Study provided the mean (SD) age for each group, but not for the overall sample. The value shown in this table was derived from the original data and weighted using a formula.

^b^Published online December 28, 2023.

^c^Reported for the full study cohort (including patients with fibromyalgia or osteoarthritis), but not for RA patients.

^d^Not reported in manuscript; data derived from clinicaltrials.gov (https://clinicaltrials.gov/study/NCT00283855).

^e^ Study enrolled patients 14 years and older but was included because reported mean (SD) ages demonstrated that the majority of participants were adults.

AS, ankylosing spondylitis; DMARD, disease-modifying antirheumatic drug; HR-QoL, health-related quality of life; NR, not reported; PsA, psoriatic arthritis; RA, rheumatoid arthritis; SD, standard deviation; SLE, systemic lupus erythematosus; SpA, spondyloarthritis; USA, United States of America, wk, week.

Twelve of the 15 included studies investigated individuals with RA (Table 3). Ten of these studies included only patients with RA, one involved individuals with RA or SLE, and one enrolled individuals with RA, spondyloarthritis (SpA), or PsA. In addition to the study of patients with RA or SLE, two other studies focused only on people with SLE. One study included individuals with ankylosing spondylitis (AS) (Table 3).

Digital interventions included the use of websites/internet programs in nine studies, mobile apps in five studies, and one study that was available as both a web app and a mobile app (Table 3). In four of the studies, the intervention included a non-digital component, such as individual counseling or telephone support (39, 40, 43, 45). The durations of the interventions varied widely, ranging from a minimum of six weeks to a maximum of one year. The control group received different kinds of care across the 15 studies, most frequently standard care (12 studies), in some cases with additional features such as gift cards or a written exercise program and recommendations. In five studies the control groups received the intervention after the RCT was completed (delayed intervention) (Table 3).

The study by Lorig et al. (41) included patients with RA, osteoarthritis, and fibromyalgia. Because the focus of this SLR was on IRDs, only analyses relevant to patients with RA were included; these data were presented in the original publication as subgroup analyses. The RCT conducted by Song et al. (44) was included despite initially meeting the exclusion criterion of involving participants younger than 18 years. This study was retained because the reported mean ages and standard deviations demonstrated that the majority of participants were significantly older than 14 [mean (SD) age of total sample 29.9 (8.23) years, intervention group 30.8 (8.82) years, and control group 29.1 (7.58) years] (44).

#### 3.2.2 Per-protocol analyses and adjusted models

The study by Allen et al. (34) was challenged by the fact that only 50% (15/30) of the intervention group (IG) actually used the digital intervention in this study (PainTRAINER program). In addition to examining the overall IRD cohort, this study also evaluated the subgroup that logged onto the program, which they characterized as “PainTRAINER users” (PTU). Four other studies presented per protocol (PP) analyses in addition to intention-to-treat (ITT) analyses (35, 36, 42, 45). In three of these studies, outcomes that were not significantly impacted by the digital intervention in the ITT analysis showed significant between-group differences in the PP analysis (35, 36, 45). In this review, both the ITT and PP results are presented for studies that included these analyses.

In addition to presenting unadjusted differences between IG and control, Li et al. (39) also used a generalized linear mixed-effect model (GLMM) to evaluate the intervention effect. GLMM models were adjusted for sex and accounted for data missing at random without the need to perform imputations. For one of the outcomes evaluated in this study, there was a difference between IG and control in the GLMM but not in the unadjusted data. Both unadjusted and GLMM data are presented for the Li et al. study (39).

### 3.3 Assessment of study quality

Each study was evaluated individually with the CASP checklist (Supplementary file S2). Overall, most studies were methodologically sound. The most common methodological flaw was the absence of participant blinding, which can be difficult to achieve in evaluations of digital tools. Only one study, Allam et al. (33), met criteria for participant blinding by using parallel experimental groups, and six met criteria for investigator blinding (23, 34, 38, 39, 44, 45). Most did not mention whether the people analyzing the outcomes were blinded (Supplementary file S2). The other most common methodological issue was not reporting confidence intervals (six studies) or effect sizes (three studies). In the study by Ferwerda et al. (35), the intervention group was not well-balanced with the control group with respect to mood and certain physical ability assessments.

### 3.4 Outcomes of relevance

The most frequent primary outcomes evaluated in the 15 studies was various aspects of self-management/self-care/self-efficacy (*n* = 11), including six studies that evaluated physical activity (interventions to encourage exercise and other physical activities), and functional impairment (*n* = 11) (Table 3). Pain was evaluated in ten studies, and depression/anxiety was assessed in six (all six evaluated depression and three evaluated anxiety, including one study that used a composite measure for depression and anxiety). For secondary outcomes, disease activity (*n* = 7) and fatigue (*n* = 7) were the most frequent outcomes assessed.

Table 4 shows a summary of evaluated outcomes and significant differences between the IG and control group. The following sections discuss each of the primary outcomes individually and summarize the secondary outcomes. An alternative presentation showing all relevant outcomes reported by each study is presented in Supplementary file S3.

**Table 4:** Summary of significant differences in favor of digital interventions vs. control in the included studies.

Study	Primary outcomes for SLR				Secondary outcomes for SLR				
	Self-management	Pain	Depression/ anxiety	Functional impairment	Disease activity	Work productivity	Medication adherence	QoL	Fatigue
Allam [2015] (33)	x								
Allen [2021] (34)		x	x	x				x	x
Ferwerda [2017] (35)	x	x	x	x	x			x	x
Khan [2020] (36)		x		x					x
Knudsen [2024] (37)	x				x		x	x	
Kurt [2024] (38)	x	x	x	x	x				
Li [2025] (39)	x	x	x		x				x
Li [2020] (40)	x	x	x						x
Lorig [2008] (41)	x	x		x		x			x
Pouls [2022] (42)				x	x		x		
Rodríguez Sánchez-Laulhé [2022] (23)		x		x (hand function)		x			
Shigaki [2013] (43)	x	x	x	x				x	
Song [2022] (44)	x			x	x				
van den Berg [2006] (45)	x			x	x			x	
Zuidema [2019] (46)	x	x		x					x

x = evaluated outcome. Blue shading indicates a significant difference between the intervention and control groups for at least one analysis, including per-protocol analyses.

QoL, quality of life; SLR, systematic literature review.

### 3.5 Primary outcomes

#### 3.5.1 Self-management, including physical activity

For self-management outcomes, we included interventions designed to improve self-empowerment, self-care, self-efficacy, self-management, patient activation, and physical activity (fitness outcomes such as time spent exercising or fitness tracker data) (Table 5). These studies had a broad spectrum of results. Of the eleven studies that evaluated self-management, six reported a significant difference in favor of the IG, including three that found significant improvements in self-efficacy with digital interventions (37, 43, 44). Two of the six studies that evaluated physical activity reported a positive result associated with the intervention (33, 44), but four did not (38–41).

**Table 5:** Results of digital interventions on self-management in IRD patients.

First author [pub year] (ref number)	Intervention	Duration	Assessment	Results
Allam [2015] (33)	Website ONESELF 1. Informational sections only 2. Informational + social support 3. Informational + gaming 4. Informational, social support, and gaming sections	16 wks	Self-empowerment: 12-item scale	Significant increase in Group 2 and Group 3 vs. control over time Group 2 β = 2.59, *p* = 0.03 Group 3 *β* = 2.29, *p* = 0.05
			Physical activity: Exercise behaviors scale	Significant increase in Group 4 for mean minutes spent on exercise Group 4 *β* = 3.39, *p* = 0.02
Ferwerda [2017] (35)	Internet-based cognitive behavioral intervention	9 to 65 wks (mean [SD], 26 [12] wks)	Self-care: Impact of rheumatic diseases on general lifestyle self-care scale	No significant between-group difference in ITT (*p* = 0.19) or PP analyses (*p*-value NR)
Knudsen [2024] (37)	Web-based digital patient education program regarding disease-specific knowledge	1 year	Self-efficacy: Rheumatoid arthritis self-efficacy	Significant improvement for IG vs. control from baseline to 12 months: Unadjusted mean (95% CI) difference: −3.65 (−7.25, −0.05), *p* = 0.047 Adjusted^a^ mean (95% CI) difference: −4.34 (−8.17, −0.51), *p* = 0.026
Kurt [2024] (38)	Mobile app (Mida Rheuma) with individualized lifestyle counselling	12 wks	Physical activity: Physical activity, excercise, and sport questionnaire	Neither the IG nor control showed a significant change over 12 wks in weekly time spent on physical activity or in exercise questionnaire scores (between group *p*-values NR)
Li [2025] (39)	Web/mobile app OPERAS	26 wks	Self-management: PAM-13	Significant improvement for IG vs. control in unadjusted and GLMM analyses at 27 wks Unadjusted mean (95% CI) difference: 6.2 (1.3, 11.1), *p* < 0.05 (exact *p*-values NR) GLMM intervention effect: 5.3 (2.0, 8.7), *d* = 0.39, *p* ≤ 0.001
			Physical activity: SenseWear Mini	No significant between-group difference in time spent in moderate/vigorous physical activity (p-value NR)
Li [2020] (40)	Fitness tracker (Fitbit) and web-based app FitViz	8 wks	Physical activity: SenseWear	No significant between-group difference vs. control at 9 wks for in time spent in moderate/vigorous physical activity (*p* = 0.06)
Lorig [2008] (41)	Internet-based arthritis self-management program	6 wks	Physical activity: Minutes/wk. of aerobic exercise and stretching/ strength exercise	No significant between-group differences over 1 year in aerobic exercise (*p* = 0.963) or stretching and strength exercise (*p* = 0.877)
Shigaki [2013] (42)	Website RAHelp	10 wks	Self-efficacy: Arthritis self-efficacy scale	Significant improvement for IG vs. control immediately post-intervention and at 9 month follow-up Post-intervention: ES = 0.92 (*p* < 0.001) 9 months: ES = 0.92 (*p* < 0.001) ^b^
Song [2022] (43)	Mobile social networking app “WeChat”	12 wks	Self-efficacy: arthritis self-efficacy scale-8	Significant improvement for IG vs. control posttest (week 12) Mean (SD) of 7.60 (1.50) for IG vs. 6.41 (2.04) for control (*p* < 0.001)
van den Berg [2006] (45)	Website “cybertraining.nl” individualized physical activity program	1 year	Physical fitness: Participant’s report of physical activity at moderate intensity for ≥30 mi*n* ≥ 5 days/wk. or at vigorous intensity ≥20 mi*n* ≥ 3 days/ wk	Significant differences for IG vs. control in ITT analyses for most timepoints evaluated ≥30 min moderate activity ≥5 days/wk. 6 months: 38% vs. 22% (*p* = 0.041) 9 months: 35% vs. 11% (*p* < 0.001) 12 months: 26% vs. 15% (*p* = 0.120) ≥20 min vigorous activity ≥3 days/wk. 6 months: 35% vs. 13% (*p* = 0.002) 9 months: 40% vs. 14% (*p* = 0.001) 12 months: 34% vs. 10% (*p* < 0.001) More favorable results for the IG observed in PP analyses, resulting in statistically significant differences in moderate activity at 12 months (*p* = 0.002) and vigorous activity at 3 months (*p* = 0.036)
Zuidema [2019] (46)	Web-based self-management program	1 year	Self-management: PAM-13	No significant between-group difference at 6 months (*p* = 0.44) or 12 months (*p* = 0.93)
			Self-management: self-management ability scale	No significant between-group difference at 6 months (*p* = 0.72) or 12 months (*p* = 0.43)
			Self-efficacy: rheumatoid arthritis self-efficacy	No significant difference between groups at 6 months (*p* = 0.16) or 12 months (*p* = 0.81)

Blue shading indicates a statistically significant improvement (*p* ≤ 0.05) compared with the control group in at least one subgroup.

Exact *p*-values and/or effect size coefficients are reported as presented in the full text of the referenced publication.

^a^Adjusted for baseline age, sex, educational level, Disease Activity Score-28 joints, and site.

^b^Effect size coefficients not reported.

ES, effect size; IG, intervention group; GLMM, Generalized Linear Mixed-effect Model; IRGL, Impact of Rheumatic Diseases on General Lifestyle; ITT, intention to treat; min, minutes; NR, not reported; OT, occupational therapy; PAM, Patient Activation Measure: PP, per protocol; PT, physical therapy; pub, publication; RASE, Rheumatoid Arthritis Self-Efficacy; ref, reference; wk, week.

#### 3.5.2 Pain

Pain outcomes in the included studies also showed mixed results (Table 6). Five of the ten studies showed improvements in pain in the IG vs. control, including one study of the “CareHand” app in which overall pain intensity was similar between the two groups, but significant improvements were observed in pain in the hands/wrists (23). Longer durations for interventions did not appear to result in a greater likelihood of positive pain outcomes, as studies with the longest durations (one year, 9 to 65 weeks, and 26 weeks) failed to show significant improvements in pain.

**Table 6:** Results of digital interventions on reduction of pain in IRD patients.

First author [pub year] (ref number)	Intervention	Duration	Assessment	Results
Allen [2021] (34)	Internet-based training on pain coping skills (PainTRAINER) Only 50% of IG (15/30) logged into program [PainTRAINER users (PTU)]	6 wks	PROMIS pain interference (form 6a) and catastrophizing domain of the coping strategies questionnaire	Improvements from BL to 9 wks in pain interference for IG and PTU vs. control (mean change of-2.6 and-3.9 vs-1.7; *d* = −0.12 for IG and *d* = −0.30 for PTU) Smaller increase from BL in pain catastrophizing for IG vs. control (mean change of 2.3 vs. 3.6; *d* = −0.16), decrease from BL for PTU (mean change of-0.9 vs. 3.6; *d* = ˗0.60)
Ferwerda [2017] (35)	Internet-based cognitive behavioral intervention	9 to 65 wks (mean [SD], 26 [12] wks)	Impact of rheumatic diseases on general lifestyle pain scale	No significant between-group difference (*p* = 0.35)
Khan [2020] (36)	Mobile smartphone app for lifestyle activities and symptoms	16 wks	Brief pain inventory-short form pain severity and pain interference domains	Significant improvements for IG vs. control at 16 wks in change from BL for pain severity and pain interference in PP analysis, but not ITT (*p* = 0.73 and 0.31) PP pain severity: between-group difference = −1.9 (*p* = 0.049) PP pain interference: between-group difference = −2.5 (*p* = 0.02)
Kurt [2024] (38)	Mobile app (Mida Rheuma) with individualized lifestyle counselling	12 wks	SF36 bodily pain	No significant changes over time in IG (*p* = 0.2) or control group (*p* = 0.8)
Li [2025] (39)	Web/mobile app OPERAS and fitness tracker (Fitbit)	26 wks	McGill Pain questionnaire-short form	No significant between-group difference in unadjusted or GLMM analyses (*p* > 0.05) (exact p-values NR)
Li [2020] (40)	Fitness tracker (Fitbit) and web-based app FitViz	8 wks	McGill Pain questionnaire-short form	Significant mean difference at 9 wks for IG compared with control: −2.45 (95% CI-4.78, −0.13; *p* = 0.04)
Lorig [2008] (41)	Internet-based arthritis self-management program	6 wks	Visual numeric scales (self-designed)	Significant improvement for IG vs. control over 1 year Mean SD change: −0.514 (2.79) for IG vs-0.069 (1.69) for control, *p* = 0.04
Rodríguez Sánchez-Laulhé [2022] (23)	Mobile CareHand app	12 wks	VAS for self-reported pain intensity	No significant between-group differences at month 1 (*p* = 0.20), month 3 (*p* = 0.87), or month 6 (*p* = 0.65)
			Michigan hand outcome questionnaire pain subscale	Significant improvements in pain in hands/wrists for IG vs. control and significant time-group effect (*p* < 0.001) Month 3 mean (95% CI) difference: −35.08 (−50.54, −19.62), *p* < 0.001 Month 6 mean (95% CI) difference: −26.06 (−39.69, −12.42), *p* < 0.001
Shigaki [2013] (43)	Website RAHelp	10 wks	Pain/interference due to pain in past 4 wks: AIMS2	No significant between-group differences immediately post-intervention (*p* = 0.07) or at 9-month follow-up (*p* = 0.34)
			Pain today: RADAR	No significant between-group differences immediately post-intervention (*p* = 0.24) or at 9-month follow-up (*p* = 0.58)
Zuidema [2019] (46)	Web-based self-management program	1 year	Numeric rating scale for pain today and pain in last 2 wks	Pain today: no significant between-group differences at 6 months (*p* = 0.97) or 12 months (*p* = 0.13) Pain in last 2 wks: no significant between-group differences at 6 months (*p* = 0.97) or 12 months (*p* = 0.60)

Blue shading indicates a statistically significant improvement (*p* ≤ 0.05) compared with the control group in at least one subgroup or an effect size (Cohen’s *d*) ≥ 0.3 for studies that did not present *p*-values.

Exact *p*-values and/or effect size coefficients are reported as presented in the full text of the referenced publication.

BL, baseline; CI, confidence interval; GLMM, Generalized Linear Mixed-effect Model; IG, intervention group; ITT, intention to treat; PP, per protocol; PROMIS, Patient-reported Outcomes Measurement Information System; pub, publication; PTU, PainTRAINER users; ref., reference; RA, rheumatoid arthritis; SD, standard deviation; SF36, 36-item Short-Form Survey; VAS, visual analog scale; wk., week.

#### 3.5.3 Symptoms of depression and anxiety

Three of the six (50%) studies that evaluated mood disorder observed a positive effect with digital interventions (Table 7). Three of the studies evaluating depressive symptoms alone and one of the studies evaluating anxiety symptoms alone reported improvements in patients in the IG vs. control. The only two studies with interventions >12 weeks showed positive results (35, 39), suggesting that longer durations may be beneficial for psychological health outcomes.

**Table 7:** Results of digital interventions on reducing symptoms of depression and/or anxiety in IRD patients.

First author [pub year] (ref number)	Intervention	Duration	Assessment	Results
Allen [2021] (34)	Internet-based training on pain coping skills (PainTRAINER) Only 50% of IG (15/30) logged into program (PainTRAINER users [PTU])	6 wks	Depression: PROMIS-29 depression domain	Moderate improvements from BL to 9 wks for IG and PTU vs. control (mean change of-3.4 and-4.1 vs-0.6; *d* = −0.32 for IG and *d* = −0.44 for PTU)
			Anxiety: PROMIS-29 anxiety domain	Similar changes from BL for IG and control (mean change of 1.4 vs. 0.4; *d* = 0.09); decrease from BL for PTU vs. control (mean change of-0.7 vs. 0.4; *d* = −0.11)
Ferwerda [2017] (35)	Internet-based cognitive behavioral intervention	9 to 65 wks (mean [SD], 26 [12] wks)	Depression: Beck Depression Index	Significant improvement for IG vs. control over 12 months in ITT and PP analyses: ITT difference between groups over time *d* = 0.54, *p* = 0.001 PP *p*-value NR
			Anxiety: impact of rheumatic diseases on general lifestyle anxiety scale	Significant improvement for IG vs. control over 12 months in ITT and PP analyses: ITT difference between groups over time *d* = 0.48, *p* = 0.001 PP *p*-value NR
Kurt [2024] (86)	Mobile app (Mida Rheuma) with individualized lifestyle counselling	12 wks	Depression and anxiety: patient health questionnaire-4	No significant differences over 12 wks for IG (*p* = 0.34) or control (*p* = 0.32)
Li [2025] (39)	Web/mobile app OPERAS and fitness tracker (Fitbit)	26 wks	Depression: patient health questionnaire-9	Significant improvement for IG vs. control in unadjusted and GLMM analyses at 27 wks: Unadjusted: mean (95% CI) difference-1.8 (−3.3, −0.2), *p* < 0.05 (exact p-value NR) GLMM intervention effect: −1.3 (−2.3, −0.3), *d* = 0.30, *p* = 0.01
Li [2020] (40)	Fitness tracker (Fitbit) and web-based app FitViz	8 wks	Depression: patient health questionnaire-9	No significant between-group difference at 27 wks (*p* ≥ 0.05) (exact *p*-value NR)
Shigaki [2013] (43)	Website RAHelp	10 wks	Depression: center for epidemiologic studies depression scale	No significant between-group difference immediately post-intervention (*p* = 0.14) or at 9-month follow-up (*p* = 0.14)

Blue shading indicates a statistically significant improvement (*p* ≤ 0.05) compared with the control group in at least one subgroup or an effect size (Cohen’s *d*) ≥ 0.3 for studies that did not present *p*-values.

Exact *p*-values and/or effect size coefficients are reported as presented in the full text of the referenced publication.

BL, baseline; CI, confidence interval; GLMM, Generalized Linear Mixed-effect Model; IG, intervention group; NR, not reported; PROMIS, Patient-reported Outcomes Measurement Information System; pub, publication; PTU, PainTRAINER users; ref, reference; wk, week.

#### 3.5.4 Functional impairment

Functional impairment assessments included outcomes for non-disease-specific (e.g., Short-Form 36 physical function subscale, Health Assessment Questionnaire disability index) and disease-specific (e.g., Bath Ankylosing Spondylitis Functional Index) functional ability; many of the assessments involved the ability to perform activities of daily living. Only three of eleven studies showed a positive effect on functional impairment in patients receiving digital interventions (Table 8), and one of these positive studies was specifically focused on hand function (23).

**Table 8:** Results of digital interventions on functional impairment in IRD patients.

First author [pub year] (ref number)	Intervention	Duration	Assessment	Results
Allen [2021] (34)	Internet-based training on pain coping skills (PainTRAINER) Only 50% of IG (15/30) logged into program [PainTRAINER users (PTU)]	6 wks	PROMIS-29 physical function domain	Improvements from BL to 9 wks for IG and PTU vs. control (mean change of-3.8 and-4.0 vs-0.6; *d* = −0.56 for IG and *d* = −0.55 for PTU)
Ferwerda [2017] (35)	Internet-based cognitive behavioral intervention	9 to 65 wks [mean (SD), 26 (12) wks]	Composite score of pain and fatigue	No significant between-group difference in change over 12 months (*p* = 0.15)
Khan [2020] (36)	Mobile smartphone app for lifestyle activities and symptoms	16 wks	Lupus quality of life physical health domain	Significant difference in PP analysis at 16 wks, but not in ITT (*p* = 0.88) PP between-group difference: 14.1 (*p* = 0.49)
Kurt [2024] (38)	Mobile app (Mida Rheuma) with individualized lifestyle counselling	12 wks	Short Form 36 physical function subscale	No significant differences over 12 wks for IG (*p* = 0.60) or control (*p* = 0.90)
Lorig [2008] (41)	Internet-based arthritis self-management program	6 wks	Health assessment questionnaire-disability index	No significant difference over 1 year for IG vs. control (*p* = 0.85)
Pouls [2022] (42)	Mobile game-based intervention	12 wks	Health assessment questionnaire-disability index	No significant between-group differences at 3 months (*p* values NR)
Rodríguez Sánchez-Laulhé [2022] (23)	Mobile CareHand app	12 wks	Michigan hand outcome questionnaire total score	Significant improvement in overall hand function for IG vs. control at 3 and 6 months and significant time-group effect (*p* < 0.001) Mean (95% CI) difference between groups 3 months: 16.86 (8.70, 25.03), *p* = 0.001 6 months: 17.21 (4.78 to 29.63), *p* = 0.007
Shigaki [2013] (43)	Website RAHelp	10 wks	Arthritis impact measurement scales 2 physical scale	No significant between-group differences immediately post-intervention (*p* = 0.065) or at 9-month follow-up (*p* = 0.16)
Song [2022] (44)	Mobile social networking app “WeChat”	12 wks	Bath ankylosing spondylitis functional index	No significant between-group difference in median physical function at posttest (wk 12) (*p* = 0.08)
van den Berg [2006] (45)	Website “cybertraining.nl” individualized physical activity program	1 year	Health assessment questionnaire-disability index	No significant between-group difference over 12-months (*p* = 0.41)
Zuidema [2019] (46)	Web-based self-management program	1 year	Short Form-36 general health status	No significant between-group difference at 6 months (*p* = 0.40) or 12 months (*p* = 0.96)

Blue shading indicates a statistically significant improvement (*p* ≤ 0.05) compared with the control group in at least one subgroup or an effect size (Cohen’s *d*) ≥ 0.3 for studies that did not present *p*-values.

Exact *p*-values and/or effect size coefficients are reported where presented in the full-text of the referenced publication.

BL, baseline; CI, confidence interval; GLMM, generalized linear mixed-effect model; IG, intervention group; NR, not reported; pub, publication; PTU, PainTRAINER users; ref, reference; wk, week.

### 3.6 Secondary outcomes

Analyses of the secondary outcomes identified for this SLR found good effects for digital interventions in some areas, but not others (Table 4; Supplementary file S4). Neither of the two studies involving medication adherence and only two of the seven studies that evaluated disease activity found differences between IG vs. control, but both studies that assessed work-related outcomes (23, 41) reported improvements with digital interventions. Three of five studies that assessed QoL found improvements for IG vs. control; in one study, the improvement was observed in the PP analysis and not in the ITT analysis (35). Two of seven studies of fatigue also found improvements.

## 4 Discussion

The goal of this SLR was to identify the relevance of digital tools for improving self-management and related outcomes in individuals with IRDs. Data from the 15 included studies, all of which were RCTs, reveal a mixed yet promising landscape for digital tools aimed at improving the health status of individuals with IRDs by enhancing self-management and self-care activities. While several objectives demonstrated significant benefits, others showed negligible effects, underscoring the different approaches to designing digital interventions and identifying suitable outcome measures in a heterogeneous patient population. This variability complicates direct comparisons but highlights the potential for these low-cost, scalable interventions to complement traditional care. Within a given study, the digital intervention typically affected some outcomes but not others, suggesting that improvement of disease management through the use of digital tools may not apply to all facets of IRDs.

The majority of studies that evaluated self-management outcomes not related to physical activity [5/7 (71.4%)] reported significant improvements in assessments of self-care, self-empowerment, self-efficacy, or patient activation. The two that did not were both web-based interventions that were primarily educational in nature (35, 46). It is possible that the lack of engagement fostered by these programs may have contributed to the negative findings. Physical activity levels showed an improvement in two studies (33, 45), but not in four others. Pain outcomes also revealed a broad spectrum of results. Half (5/10) of the studies highlighted significant pain reductions among participants in intervention groups, supporting the potential of digital programs to address one of the most debilitating symptoms of IRDs. The evaluation of depression and anxiety similarly yielded mixed results. While three of six (50%) interventions demonstrated significant reductions in depression symptoms (34, 35, 39), the other three found no substantial improvements (38, 40, 43). Of the two studies that specifically evaluated anxiety outcomes, one found a significant improvement (35) while the other did not (34). The limited success of digital interventions in managing mood disorders may relate to the complexity of addressing psychological well-being in the context of IRDs, as these conditions often require multi-faceted, long-term interventions that go beyond the scope of most digital programs. Furthermore, many of the interventions had a duration of only a few weeks, and longer time periods may be required for changes in psychological health. A study of cognitive behavioral therapy in medication-free patients with major depressive disorder found that depressive symptoms continued to improve over three months of therapy (47). Only three of the eleven studies that evaluated functional impairment found improvements for IG vs. control, and one of these was specifically focused on hand function (23).

With respect to the secondary outcomes identified for this SLR, only two of seven studies found a change in objective assessments of disease activity following digital interventions, which likely reflects the need for pharmaceutical approaches as the backbone for IRD disease management. Only two studies evaluated medication adherence and neither found improvements for IG vs. control. Although the lack of success with digital tools in improving medication adherence is consistent with another review of medication adherence in chronic conditions (diabetes and hypertension) (48), it should be noted that this field is one of active investigation and favorable results have been reported in some studies (49, 50). Evaluations of QoL and fatigue were positive in some studies, but not in others. Both studies that evaluated work productivity found improvements for IG vs. control (23, 41). Given the profound impact of IRDs on work participation and productivity (51, 52), these observed benefits suggest the use of digital tools to improve work outcomes deserves further study.

In addition to the seven studies that reported objective assessments of disease activity, the study by Rodríguez Sánchez-Laulhé et al. (23) evaluated the objective measures of hand grip strength and pinch strength, neither of which were affected by the digital intervention. The other studies (7/15) focused solely on patient-reported outcomes and did not include objective assessments. This approach allows large amounts of data to be collected more conveniently, but has the limitation of lacking data on the effect of the intervention on outcome measures such as joint counts or laboratory markers.

The heterogeneity of included studies poses a significant challenge to drawing overarching conclusions. Interventions varied widely in their design, content, and delivery methods, ranging from gamified programs to structured educational modules. This variability makes direct comparisons difficult and highlights the importance of tailoring interventions to meet the specific needs of diverse patient populations. Although all of the studies involved patients with IRDs, care needs may vary within this group of diseases depending on the specific disease and its characteristics. For instance, individuals with RA may require tools focused on joint protection and pain management, while those with SLE may benefit more from tools that address the systemic nature of the disease and its psychological impacts. The potential differences in response to digital tools across IRDs is supported by subgroup analyses in the study by Li et al. (40), which showed significant improvements in physical activity and pain for patients with RA, but not those with SLE. Moreover, some studies included highly specific participant groups, which may have influenced the results. For example, the study by Rodríguez Sánchez-Laulhé et al. specifically targeted patients with RA affecting the hands, wrists, or fingers for at least two years who reported current pain and disability (23). Selecting individuals with specific needs may allow more precise tailoring of the intervention, while focusing on patients who are already experiencing significant pain and functional limitations may provide more opportunity for improvement compared with studies of patients with better-controlled symptoms, potentially increasing the likelihood of a positive outcome.

There were also marked variations in the assessment tools used, even for a single symptom such as pain or fatigue. The potential effects of different assessments is illustrated by QoL evaluations in the study by Allen et al., which did not find a significant difference between IG and control in patients with SLE using the Patient Reported Outcomes Measurement Information System (PROMIS)-29, a general QoL assessment tool, but did observe a significant difference with the more disease-specific LupusPRO tool (34).

For some studies, findings may have been influenced by the duration of the digital intervention. The intervention lasted one year in three of the studies (37, 45, 46), but most of the others had intervention durations of 6 to 12 weeks. Longer studies may be more likely to capture the long-term effects of interventions on certain symptoms, such as chronic pain and fatigue, but also have the challenge of maintaining user engagement once the “novelty” of the tool has worn off. In this SLR no clear pattern was observed between the length of the intervention and the likelihood of positive outcomes. This suggests that other factors, such as the nature of the intervention, the content provided, and participant engagement, may play a more significant role in determining the effectiveness of the intervention than the duration alone.

Additional factors could have also influenced the presented results. The potential for “contamination” in control groups is a concern; in many studies it could not be guaranteed that participants in the control groups refrained from accessing alternative digital tools or platforms, which could have diluted observed between-group differences. As for all interventions, adherence is an important issue for digital tools. In one study, results of the intervention group were further divided into results obtained with patients documented to have logged onto the website (34). Several of the evaluated outcomes were positive for the user group, but not for the IG as a whole. Other studies reported PP analyses in addition to ITT analyses including all IG patients (35, 36, 42, 45), and this resulted in modest differences in significant outcomes in three of them (35, 36, 45). A better understanding of why patients do—and do not—choose to engage in digital interventions may be an important factor in integrating these tools into everyday use.

Given the variability among studies, our literature review was not designed to address which type of digital intervention (web-based, mobile app, or fitness tracker) was more effective. In addition, some interventions had non-digital components (telephone support, counseling sessions, etc.) (39, 40, 43, 45). Thus, it remains debatable whether the digital intervention alone would have resulted in similar results.

Despite the variability of the results, the findings of this review are largely encouraging, particularly given the low cost and scalability of digital interventions. Automated, internet-based programs hold significant potential for widespread dissemination and use, especially among younger, tech-savvy populations. The results of some included studies, such as the one by Ferwerda et al. (33), indicate that digital interventions are largely consistent with outcomes from face-to-face tailored cognitive behavioral therapy trials in patients with RA, thus suggesting that the online applications appear to be an effective platform for delivering this type of intervention. The observed improvements in self-efficacy, pain reduction, and other outcomes, although variable, suggest that these tools can play a valuable role in complementing traditional care. The results are particularly relevant considering the large and growing numbers of individuals affected by IRDs and limited patient-care resources in rheumatology (9). Future research should prioritize standardization of intervention designs, incorporation of longer follow-up periods, and expansion of studies to more diverse populations and settings. Tailored strategies to enhance engagement among older adults and individuals with limited digital literacy will also be critical. While current evidence highlights the promise of digital interventions, addressing gaps in study design, adherence, and implementation will be essential for maximizing their impact on the self-management and overall well-being of individuals with IRDs.

Safety is another important consideration in studies of digital health applications, but this outcome is seldom investigated or reported. Safety concerns tend to fall into several general areas, including patient harm [patient deterioration due to less frequent visits, injury due to actions proposed by the health application, such as exercise movements (40), or misinformation/misunderstanding leading to harm] and data security (53). Other potential safety issues include addictive behavior or increased stress and depression related to digital monitoring (54). Future studies should endeavor to evaluate these outcomes in addition to the benefits of digital health applications.

It should be noted that this is a field of active and ongoing investigation and new digital applications are constantly being explored. In addition to the studies and interventions included in this SLR, there are other digital health applications that may be of use in patients with rheumatologic conditions that did not meet the entry criteria for this study. As an example, the RheCORD Plus patient support and information app, which supports disease documentation and self-care and was developed through the collaborative efforts of several groups, including the German Society for Rheumatologists (BDRh), is currently recruiting patients for a clinical trial (55). Albrecht et al. (56) recently reported on twelve different prescribable digital tools used in German patients with rheumatologic conditions to manage associated symptoms such as pain and insomnia. To date, no prescribable digital health applications specifically for use in patients with inflammatory arthritis have been approved. There are also other digital interventions whose trials were published after cut-off for this SLR, such as the cognitive-behavioral tool *reclarit* (25) for patients with RA, or that have not yet had publications of randomized clinical trials, including the Axia (57) and YogiTherapy (58) exercise apps for patients with axial spondyloarthritis and the RheumaBuddy app for patients with RA (59). Other digital health applications are being developed for rheumatologic conditions not included in this SLR, including systemic sclerosis. A new digital app for this condition, the SALVE app, combines photography with patient-reported assessments to track patient hand outcomes (60). A complete list of all the apps being explored in the field of rheumatology is beyond the scope of this publication, but it is important to highlight the wide breadth and rapid advancement of this area.

In addition to the previously discussed variability in study designs, this SLR has several additional limitations related to the search scope, potential biases, and generalizability of the results that should be considered when interpreting the findings. The search was conducted exclusively through the databases CINAHL, PubMed, Embase and Cochrane. Although these databases are comprehensive, it is possible that relevant studies in other databases were missed. Only published studies were included, which may introduce publication bias by limiting findings to studies with predominantly positive results. This bias arises from the higher likelihood of studies with statistically significant positive outcomes being published compared to those reporting non-significant or negative findings (61). Various digital interventions in IRDs are being intensively investigated, so after the search cut-off date other relevant studies have been published, including one by Betz et al. (25). Studies were restricted to publications in English and German, potentially omitting valuable research in other languages (62). Only studies involving digital interventions were included. Other intervention types that are potentially beneficial for self-management in people with IRDs were not considered. The selection was limited to RCTs and SLRs/meta-analyses in order to ensure a higher quality of reporting; additional relevant insights might have been found in observational studies or qualitative research. The included studies evaluated outcomes in adults and therefore these findings do not necessarily apply to individuals under 18 years of age. Additionally, some studies included in this review lack comprehensive data on confounding factors, such as comorbid conditions or variations in treatment adherence, which could influence the reported outcomes.

## 5 Conclusion

This systematic review investigated the potential and relevance of digital tools for aspects of self-management and self-care in individuals with IRDs. Overall, certain interventions demonstrated significant benefits in fatigue, pain interference, symptoms of depression, and self-efficacy outcomes. Improvements in physical activity and disease activity were evident in some studies but not consistently across all metrics. The findings underscore the value of tailored interventions, with notable effects in specific subgroups.

As the number of patients with IRDs and the complexity of care continue to increase, digital tools are likely to constitute a critical component of patient management and provide a way to bridge the gap in care for people with IRDs. Further research is warranted to explore long-term effects and optimize intervention strategies.

## Data Availability

The original contributions presented in the study are included in the article/Supplementary material. Further inquiries can be directed to the corresponding author.
