# Supplementary material for: Potential of digital applications for self-management and other outcomes in inflammatory rheumatic diseases: a systematic literature review
Source: Front Med (Lausanne). 2025 Jul 9;12:1617151. doi: 10.3389/fmed.2025.1617151 (PMC12285587; doi:10.3389/fmed.2025.1617151)
Supplement: Supplementary file 1 [file Table_1.pdf]

Supplementary Material

Supplementary File S1. Detailed search strategies for PubMed, Cochrane, Embase, and the Cumulated Index in Nursing and Allied Health Literature (CINAHL).

Search strategy for PubMed (July 10, 2024)

Sequence	Search Term	Hits
#1	arthritis, rheumatoid[mesh]	129348
#2	rheumatoid arthritis[text word]	126909
#3	chronic polyarthritis[text word]	1716
#4	rheumatoid[text word]	166470
#5	reumatoid[text word]	20
#6	rheumatic[text word]	72647
#7	reumatic[text word]	3
#8	rheumat*[text word]	253122
#9	reumat*[text word]	678
#10	arthrit*[text word]	266185
#11	artrit*[text word]	538
#12	diseas*[text word]	7859989
#13	condition*[text word]	2771320
#14	nodule*[text word]	108598
#15	Arthritis, Psoriatic[mesh]	8678
#16	psoria*[text word]	70178
#17	arthriti*[text word]	265984
#18	arthropath*[text word]	13217
#19	systemic lupus erythematosus[text word]	62127
#20	lupus erythematosus, systemic[mesh]	69115
#21	Lupus[text word]	105762
#22	lupus nephritis[mesh]	8276
#23	bechterew*[text word]	594
#24	disease[text word]	5520934
#25	Spondylarthropathies[mesh]	29297
#26	ankylos*[text word]	30311
#27	spondyl*[text word]	56926
#28	axial spondyl*[text word]	3116
#29	bekhterev*[text word]	189
#30	Marie[text word]	8968
#31	struempell*[text word]	19

Sequence	Search Term	Hits
#32	Bechterew's disease[text word]	360
#33	undifferentiated arthritis[text word]	484
#34	inflammatory arthritis[title]	2013
#35	inflammatory rheumatic [title]	915
#36	(#1 OR #2 OR #3 OR ((#4 OR #5 OR #6 OR #7 OR #8 OR #9) AND (#10 OR #11 OR #12 OR #13 OR #14)) OR #15 OR (#16 AND (#17 OR #18)) OR #19 OR #20 OR #21 OR #22 OR (#23 AND #24) OR #25 OR (#26 OR #27 OR #28) OR (#29 OR #23) OR (#30 AND #31) OR #32 OR #33) OR #34 OR #35	394872
#37	appl*[title]	446368
#38	online*[title]	45020
#39	web-bas*[title]	10846
#40	mobile[title]	30224
#41	digital[title]	60975
#42	program*[title]	248904
#43	education[title]	154594
#44	e-health[title]	1447
#45	telemedicine[title]	10710
#46	mHealth[title]	3065
#47	digital health[title]	2947
#48	online platform[title]	163
#49	internet [title]	21894
#50	mindfulness*[title]	8283
#51	relaxation[title]	27298
#52	stress-reduc*[title]	1336
#53	breath*[title]	37371
#54	forest-bathing[title]	46
#55	shinrin-yoku[title]	27
#56	self-acceptance[title]	142
#57	psycho* intervention* [title]	4643
#58	(#37 OR #38 OR #39 OR #40 OR #41 OR #42 OR #43 OR #44 OR #45 OR #46 OR #47 OR #48 OR #49 OR #50 OR #51 OR #52 OR #53 OR #54 OR #55 OR #56 OR #57)	1071204
#59	standard of care[text word]	51675
#60	gold standard [text word]	95702
#61	routine*[text word]	504284
#62	usual care[text word]	25127
#63	conventional treatment[text word]	15130
#64	standard therapy[text word]	15580
#65	placebo [text word]	257848

Sequence	Search Term	Hits
#66	clinical practice [title]	40543
#67	practice [title]	230950
#68	care [title]	640351
#69	randomiz*[text word]	1097859
#70	randomis*[text word]	143049
#71	(#59 OR #60 OR #61 OR #62 OR #63 OR #64 OR #65 OR#66 OR #67 OR #68 OR #69 OR #70)	3308092
#72	self-management[text word]	30161
#73	self-care[text word]	53948
#74	health maintenance[text word]	20317
#75	self-empowerment[text word]	286
#76	physical function*[text word]	38812
#77	physical abilities[text word]	870
#78	bodily capabilities[text word]	5
#79	pain[mesh]	477553
#80	pain measurement[mesh]	96264
#81	pain intensity[text word]	28101
#82	depression[mesh]	267347
#83	depressive disorder[mesh]	125286
#84	depressed mood[text word]	5680
#85	depressiv*[text word]	226380
#86	psychological distress[title]	7790
#87	impact of disease[text word]	1838
#88	disease impact[text word]	1610
#89	illness consequences[text word]	82
#90	disease effects[text word]	1030
#91	quality of life[mesh]	290797
#92	„health-related quality of life"[text word]	64345
#93	treatment satisfaction[text word]	4604
#94	Outcome Assessment, Health Care[mesh]	1390053
#95	Outcome and Process Assessment, Health Care[mesh]	1420323
#96	disease activity[text word]	58617
#97	patient compliance[mesh]	87173
#98	adherence [text word]	209392
#99	(#72 OR #73 OR #74 OR #75 OR #76 OR #77 OR #78 OR #79 OR #80 OR #81 OR #82 OR #83 OR #84 OR #85 OR #86 OR #87 OR #88 OR #89 OR #90 OR #91 OR #92 OR #93 OR #94 OR #95 OR #96 OR #97 OR #98)	2704496
#100	#36 AND #58 AND #71 AND #99	509

Search strategy for Cochrane (July 15, 2024)

Sequence	Search term	Hits
#1	MeSH descriptor: [Arthritis, Rheumatoid] explode all trees	8076
#2	"rheumatoid arthritis":ti,ab,kw	18420
#3	"chronic polyarthritis":ti,ab,kw	152
#4	rheumatoid:ti,ab,kw	19560
#5	reumatoid:ti,ab,kw	26
#6	rheumatic:ti,ab,kw	4887
#7	reumatic:ti,ab,kw	5
#8	rheumat*:ti,ab,kw	27542
#9	reumat*:ti,ab,kw	117
#10	arthrit*:ti,ab,kw	28596
#11	artrit*:ti,ab,kw	95
#12	diseas*:ti,ab,kw	562351
#13	condition*:ti,ab,kw	402501
#14	nodule*:ti,ab,kw	3329
#15	MeSH descriptor: [Arthritis, Psoriatic] explode all trees	737
#16	psoria*:ti,ab,kw	11656
#17	arthriti*:ti,ab,kw	28573
#18	arthropath*:ti,ab,kw	1042
#19	"systemic lupus erythematosus":ti,ab,kw	2851
#20	MeSH descriptor: [Lupus Erythematosus, Systemic] explode all trees	1606
#21	Lupus:ti,ab,kw	4364
#22	MeSH descriptor: [Lupus Nephritis] explode all trees	383
#23	bechterew*:ti,ab,kw	23
#24	disease:ti,ab,kw	479445
#25	MeSH descriptor: [Spondylarthropathies] explode all trees	1747
#26	ankylos*:ti,ab,kw	3020
#27	spondyl*:ti,ab,kw	6323
#28	axial NEXT spondyl*:ti,ab,kw	944
#29	bekhterev*:ti,ab,kw	4
#30	bechterew*:ti,ab,kw	23
#31	marie:ti,ab,kw	345
#32	struempell*:ti,ab,kw	0
#33	bechterew* NEXT disease:ti,ab,kw	14
#34	"undifferentiated arthritis":ti,ab,kw	76
#35	"inflammatory arthritis":ti	172
#36	"inflammatory rheumatic":ti	44

Sequence	Search term	Hits
#37	(#1 OR #2 OR #3 OR ((#4 OR #5 OR #6 OR #7 OR #8 OR #9) AND (#10 OR #11 OR #12 OR #13 OR #14)) OR #15 OR (#16 AND (#17 OR #18)) OR #19 OR #20 OR #21 OR #22 OR (#23 AND #24) OR #25 OR (#26 OR #27 OR #28) OR (#29 OR #30) OR (#31 AND #32) OR #33 OR #34) OR #35 OR #36	36697
#38	appl*:ti	26745
#39	online*:ti	5923
#40	web NEXT bas*:ti	3447
#41	mobile:ti	5590
#42	digital:ti	4827
#43	program*:ti	46030
#44	education:ti	16761
#45	"e health":ti	768
#46	telemedicine:ti	1338
#47	mhealth:ti	1092
#48	"digital health":ti	357
#49	"online platform":ti	19
#50	internet:ti	4593
#51	mindfulness*:ti	6062
#52	relaxation:ti	3556
#53	stress NEXT reduc*:ti	1289
#54	breath*:ti	6672
#55	"forest bathing":ti	24
#56	"shinrin yoku":ti	8
#57	"self acceptance":ti	13
#58	psycho* NEXT intervention*:ti	2638
#59	(#38 #39 OR #40 OR #41 OR #42 OR #43 OR #44 OR #45 OR #46 OR #47 OR #48 OR #49 OR #50 OR #51 OR #52 OR #53 OR #54 OR #55 OR #56 OR #57 OR #58)	95878
#60	"standard of care":ti,ab,kw	27098
#61	"gold standard":ti,ab,kw	9385
#62	routine*:ti,ab,kw	75444
#63	"usual care":ti,ab,kw	33472
#64	"conventional treatment":ti,ab,kw	6883
#65	"standard therapy":ti,ab,kw	7525
#66	placebo:ti,ab,kw	383323
#67	"clinical practice":ti	1807
#68	practice:ti	10567
#69	care:ti	54085
#70	randomiz*:ti,ab,kw	1111920
#71	randomis*:ti,ab,kw	186976

Sequence	Search term	Hits
#72	(#60 OR #61 OR #62 OR #63 OR #64 OR #65 OR #66 OR #67 OR #68 OR #69 OR #70 OR #71)	1383991
#73	"self-management":ti,ab,kw	11493
#74	"self-care":ti,ab,kw	15779
#75	"health maintenance":ti,ab,kw	868
#76	"self-empowerment":ti,ab,kw	68
#77	physical NEXT function*:ti,ab,kw	15544
#78	"physical abilities":ti,ab,kw	196
#79	"bodily capabilities":ti,ab,kw	0
#80	MeSH descriptor: [Pain] explode all trees	72817
#81	MeSH descriptor: [Pain Measurement] explode all trees	27016
#82	"pain intensity":ti,ab,kw	21810
#83	MeSH descriptor: [Depression] explode all trees	18379
#84	MeSH descriptor: [Depressive Disorder] explode all trees	16683
#85	"depressed mood":ti,ab,kw	1399
#86	depressiv*:ti,ab,kw	38381
#87	"psychological distress":ti	804
#88	"impact of disease":ti,ab,kw	327
#89	"disease impact":ti,ab,kw	234
#90	"illness consequences":ti,ab,kw	5
#91	"disease effects":ti,ab,kw	211
#92	MeSH descriptor: [Quality of Life] explode all trees	44306
#93	"health-related quality of life":ti,ab,kw	24535
#94	"treatment satisfaction":ti,ab,kw	3468
#95	MeSH descriptor: [Outcome Assessment, Health Care] explode all trees	214716
#96	MeSH descriptor: [Outcome and Process Assessment, Health Care] explode all trees	217656
#97	"disease activity":ti,ab,kw	16043
#98	MeSH descriptor: [Patient Compliance] explode all trees	15531
#99	adherence:ti,ab,kw	48913
#100	(#73 OR #74 OR #75 OR #76 OR #77 OR #78 OR #79 OR #80 OR #81 OR #82 OR #83 OR #84 OR #85 OR #86 OR #87 OR #88 OR #89 OR #90 OR #91 OR #92 OR #93 OR #94 OR #95 OR #96 OR #97 OR #98 OR #99)	436076
#101	#37 AND #59 AND #72 AND #100	543

Search strategy for Embase (July 15, 2024)

Sequence	Search term	Hits
#1	'rheumatoid arthritis'/exp	265453
#2	rheumatoid arthritis':ti	110226
#3	chronic polyarthritis':ti	1282
#4	rheumatoid:ti	121797
#5	reumatoid:ti	36
#6	rheumatic:ti	35120
#7	reumatic:ti	4
#8	rheumat*:ti	183562
#9	reumat*:ti	884
#10	arthrit*:ti	181465
#11	artrit*:ti	258
#12	diseas*:ti	1887492
#13	condition*:ti	248741
#14	nodule*:ti	34719
#15	psoriatic arthritis'/exp	32728
#16	psoria*:ti	66333
#17	arthriti*:ti	181067
#18	arthropath*:ti	6008
#19	systemic lupus erythematosus':ti	48171
#20	systemic lupus erythematosus'/exp	137005
#21	lupus:ti	83423
#22	lupus nephritis'/exp	23033
#23	bechterew*:ti	500
#24	disease:ti	1558435
#25	'spondyloarthropathy'/exp	93944
#26	ankylos*:ti	19111
#27	spondyl*:ti	44333
#28	axial spondyl*':ti	4978
#29	bekhterev*:ti	117
#30	bechterew*:ti	500
#31	marie:ti	5667
#32	struempell*:ti	17
#33	bechterew* disease':ti	32
#34	undifferentiated arthritis':ti	238
#35	inflammatory arthritis':ti	3562
#36	inflammatory rheumatic':ti	1533
#37	(#1 OR #2 OR #3 OR ((#4 OR #5 OR #6 OR #7 OR #8 OR #9) AND (#10 OR #11 OR #12 OR #13 OR #14)) OR #15 OR (#16 AND (#17 OR #18)) OR #19 OR #20 OR #21 OR #22 OR (#23 AND #24) OR #25 OR (#26 OR #27 OR #28) OR (#29 OR #30) OR (#31 AND #32) OR #33 OR #34 OR #35 OR #36)	480643

Sequence	Search term	Hits
#38	appl*:ti	516356
#39	online*:ti	53904
#40	web bas*':ti	12518
#41	mobile:ti	35108
#42	digital:ti	70809
#43	program*:ti	318310
#44	education:ti	180562
#45	e health':ti	1981
#46	telemedicine:ti	14022
#47	mhealth:ti	3080
#48	digital health':ti	3350
#49	online platform':ti	228
#50	internet:ti	26437
#51	mindfulness*':ti	9695
#52	relaxation:ti	26872
#53	stress reduc*':ti	2041
#54	breath*:ti	50419
#55	forest bathing':ti	51
#56	shinrin yoku':ti	30
#57	self acceptance':ti	153
#58	psycho* intervention*':ti	6066
#59	(#38 OR #39 OR #40 OR #41 OR #42 OR #43 OR #44 OR #45 OR #46 OR #47 OR #48 OR #49 OR #50 OR #51 OR #52 OR #53 OR #54 OR #55 OR #56 OR #57 OR #58)	1276682
#60	standard of care':ti,ab,kw	99163
#61	gold standard':ti,ab,kw	156468
#62	routine*:ti,ab,kw	734589
#63	usual care':ti,ab,kw	34992
#64	conventional treatment':ti,ab,kw	22102
#65	standard therapy':ti,ab,kw	28239
#66	placebo:ti,ab,kw	380546
#67	clinical practice':ti	55618
#68	practice:ti	287488
#69	care:ti	825451
#70	randomiz*:ti,ab,kw	1056670
#71	randomis*:ti,ab,kw	209338
#72	(#60 OR #61 OR #62 OR #63 OR #64 OR #65 OR #66 OR #67 OR #68 OR #69 OR #70 OR #71)	3321757
#73	self-management':ti,ab,kw	40997
#74	self-care':ti,ab,kw	37875
#75	health maintenance':ti,ab,kw	10127
#76	self-empowerment':ti,ab,kw	420

Sequence	Search term	Hits
#77	physical function*':ti,ab,kw	55943
#78	physical abilities':ti,ab,kw	1232
#79	bodily capabilities':ti,ab,kw	7
#80	pain/exp	1824300
#81	pain measurement'/exp	35044
#82	pain intensity':ti,ab,kw	38610
#83	depression'/exp	691191
#84	depressive disorder'/exp	691191
#85	depressed mood'	8014
#86	depressiv*:ti,ab,kw	219068
#87	psychological distress':ti	8980
#88	impact of disease':ti,ab,kw	3559
#89	disease impact':ti,ab,kw	2727
#90	illness consequences':ti,ab,kw	118
#91	disease effects':ti,ab,kw	1452
#92	quality of life'/exp	703874
#93	health-related quality of life':ti,ab,kw	92827
#94	treatment satisfaction':ti,ab,kw	8039
#95	outcome assessment'/exp	923225
#96	treatment outcome'/exp	2701951
#97	disease activity':ti,ab,kw	114728
#98	patient compliance'/exp	202365
#99	adherence:ti,ab,kw	261042
#100	(#73 OR #74 OR #75 OR #76 OR #77 OR #78 OR #79 OR #80 OR #81 OR #82 OR #83 OR #84 OR #85 OR #86 OR #87 OR #88 OR #89 OR #90 OR #91 OR #92 OR #93 OR #94 OR #95 OR #96 OR #97 OR #98 OR #99)	5598318
#101	(#37 AND #59 AND #72 AND #100)	882

Search strategy for the Cumulated Index in Nursing and Allied Health Literature (CINAHL) (July 15, 2024)

Sequence	Search term	Hits
S1	(SU "Arthritis, Rheumatoid")	25671
S2	TI "rheumatoid arthritis"	18720
S3	TI "chronic polyarthritis"	13
S4	TI "rheumatoid"	19628
S5	TI "reumatoid"	1
S6	TI "rheumatic"	4434
S7	TI "reumatic"	0
S8	TI "rheumat*"	29716
S9	TI "reumat*"	65
S10	TI "arthrit*"	34549

Sequence	Search term	Hits
S11	TI "artrit*"	102
S12	TI "diseas*"	324989
S13	TI "condition*"	34762
S14	TI "nodule*"	5720
S15	(SU "Arthritis, Psoriatic")	3221
S16	TI "psoria*"	9763
S17	TI "arthriti*"	34521
S18	TI "arthropath*"	852
S19	TI "systemic lupus erythematosus"	6805
S20	(SU "lupus erythematosus, systemic")	10576
S21	TI "lupus"	10748
S22	(SU "lupus nephritis")	1433
S23	TI "bechterew*"	6
S24	TI "disease"	287087
S25	(SU "Spondylarthropathies")	430
S26	TI "ankylos*"	3285
S27	TI "spondyl*"	8843
S28	TI "axial spondyl*"	1186
S29	TI "bekhterev*"	3
S30	TI "bechterew*"	6
S31	TI "Marie"	1281
S32	TI "strumpell*"	2
S33	TI "Bechterew's disease"	2
S34	TI "undifferentiated arthritis"	58
S35	TI "inflammatory arthritis"	853
S36	TI "inflammatory rheumatic"	268
S37	(S1 OR S2 OR S3 OR ((S4 OR S5 OR S6 OR S7 OR S8 OR S9) AND (S10 OR S11 OR S12 OR S13 OR S14)) OR S15 OR (S16 AND (S17 OR S18)) OR S19 OR S20 OR S21 OR S22 OR (S23 AND S24) OR S25 OR (S26 OR S27 OR S28) OR (S29 OR S30) OR (S31 AND S32) OR S33 OR S34) OR S35 OR S36	58820
S38	TI "appl*"	69623
S39	TI "online*"	29512
S40	TI "web bas*"	5859
S41	TI "mobile"	12640
S42	TI "program*"	143008
S43	TI "education"	106256
S44	TI "e health"	1032
S45	TI "telemedicine"	5348
S46	TI "mhealth"	1522
S47	TI "digital health"	1660
S48	TI "online platform"	57

Sequence	Search term	Hits
S49	TI "internet"	14519
S50	TI "mindfulness*"	5954
S51	TI "relaxation"	3065
S52	TI "stress reduc*"	978
S53	TI "breath*"	12236
S54	TI "forest bathing"	22
S55	TI "shinrin yoku"	12
S56	TI "self acceptance"	84
S57	TI "psycho* intervention*"	2930
S58	(S38 OR S39 OR S40 OR S41 OR S42 OR S43 OR S44 OR S45 OR S46 OR S47 OR S48 OR S49 OR S50 OR S51 OR S52 OR S53 OR S54 OR S55 OR S56 OR S57)	391071
S59	TX "standard of care"	38575
S60	TX "gold standard"	47088
S61	TX "routine*"	285695
S62	TX "usual care"	21332
S63	TX "conventional treatment"	6568
S64	TX "standard therapy"	6014
S65	TX "placebo"	113946
S66	TI "clinical practice"	19909
S67	TI "practice"	155524
S68	TI "care"	466209
S69	TX "randomiz*"	462196
S70	TX "randomis*"	113100
S71	(S59 OR S60 OR S61 OR S62 OR S63 OR S64 OR S65 OR S66 OR S67 OR S68 OR S69 OR S70)	1360095
S72	TX "self-management"	39347
S73	TX "self-care"	92953
S74	TX "health maintenance"	14575
S75	TX "self-empowerment"	809
S76	TX "physical function*"	35202
S77	TX "physical abilities"	2194
S78	TX "bodily capabilities"	12
S79	(SU "pain")	234782
S80	(SU "pain measurement")	53251
S81	TX "pain intensity"	20498
S82	(SU "depression")	153245
S83	(SU "depressive disorder")	18153
S84	"depressed mood"	80372
S85	TX "depressiv*"	94227
S86	TI "psychological distress"	4755
S87	TX "impact of disease"	2451

Sequence	Search term	Hits
S88	TX "disease impact"	1242
S89	TX "illness consequences"	159
S90	TX "disease effects"	814
S91	(SU "quality of life")	185628
S92	TX "health-related quality of life"	49791
S93	TX "treatment satisfaction"	2625
S94	(SU "Outcome Assessment, Health Care")	256985
S95	(SU "Outcome and Process Assessment, Health Care")	164544
S96	TX "disease activity"	17763
S97	(SU "patient compliance")	35748
S98	TX "adherence"	134171
S99	(S72 OR S73 OR S74 OR S75 OR S76 OR S77 OR S78 OR S79 OR S80 OR S81 OR S82 OR S83 OR S84 OR S85 OR S86 OR S87 OR S88 OR S89 OR S90 OR S91 OR S92 OR S93 OR S94 OR S95 OR S96 OR S97 OR S98)	853137
S100	S37 AND S58 AND S71 AND S99	177
