# Supplementary material for: Potential of digital applications for self-management and other outcomes in inflammatory rheumatic diseases: a systematic literature review
Source: Front Med (Lausanne). 2025 Jul 9;12:1617151. doi: 10.3389/fmed.2025.1617151 (PMC12285587; doi:10.3389/fmed.2025.1617151)
Supplement: Supplementary file 2 [file Table_2.pdf]

Supplementary Material

Supplementary File S2 Critical Appraisal Skills Program (CASP) assessment of included studies

CASP checklist for RCTs (see below for details of assessments)

**Study**	**1**	**2**	**3**	**4a**	**4b**	**4c**	**5**	**6**	**7**	**8**	**9**	**10**	**11**
Allam (2015)	Y	Y	Y	Y	N	C	Y	Y	Y	N	Y	Y	Y
Allen (2021)	Y	Y	Y	N	Y	C	Y	Y	Y	N	Y	Y	Y
Ferwerda (2017)	Y	Y	Y	N	N	C	N	Y	Y	N	Y	Y	Y
Khan (2020)	Y	Y	Y	N	N	C	Y	Y	Y	N	N	Y	Y
Knudsen (2024)	Y	Y	Y	N	N	C	Y	Y	Y	Y	N	Y	Y
Kurt (2024)	Y	Y	C	N	Y	C	Y	Y	Y	Y	Y	Y	Y
Li (2025)	Y	Y	Y	N	Y	Y	Y	Y	Y	Y	Y	Y	Y
Li (2020)	Y	Y	Y	N	N	Y	Y	Y	Y	Y	Y	Y	Y
Lorig (2008)	Y	C	Y	N	N	C	Y	Y	Y	N	Y	Y	Y
Pouls (2022)	Y	Y	Y	N	N	Y	Y	Y	Y	Y	N	Y	Y
Rodríguez Sánchez-Laulhé (2022)	Y	Y	Y	N	Y	C	N	Y	Y	Y	Y	Y	Y
Shigaki (2013)	Y	C	Y	N	C	C	Y	Y	Y	N	Y	Y	Y
Song (2022)	Y	Y	Y	N	Y	C	Y	Y	Y	Y	Y	Y	Y
van den Berg (2006)	Y	Y	Y	N	Y	C	Y	Y	Y	Y	Y	Y	Y
Zuidema (2019)	Y	Y	Y	N	N	C	Y	Y	Y	Y	Y	Y	Y

CASP questions: Did the study address a clearly formulated research question? 2: Was the assignment of participants to interventions randomised? 3: Were all participants who entered the study accounted for at its conclusion? 4: (a) Were the participants 'blind' to intervention they were given? (b) Were the investigators 'blind' to the intervention they were giving to participants? (c) Were the people assessing/analysing outcome/s 'blinded'? 5: Were the study groups similar at the start of the randomised controlled trial? 6: Apart from the experimental intervention, did each study group receive the same level of care (that is, were they treated equally)? 7: Were the effects of intervention reported comprehensively? 8: Was the precision of the estimate of the intervention or treatment effect reported? 9: Do the benefits of the experimental intervention outweigh the harms and costs? 10: Can the results be applied to your local population/in your context? 11: Would the experimental intervention provide greater value to the people in your care than any of the existing interventions?

Y, Yes; N, No, C; Can't tell

Appraisal summary of CASP checklist for RCTs

Study	Positive/<br><br>Methodologically sound	Negative/Relatively poor methodology	Unknowns
Allam (2015)	1, 2, 3, 4a, 5, 6, 7, 9, 10, 11	4b – the investigators were not blinded to allocation into intervention and control group<br><br>8 – no confidence intervals were reported	4c – it is not mentioned whether the people analysing the outcomes were blinded
Allen (2021)	1, 2, 3, 4b, 5, 6, 7, 9, 10, 11	4a – participants were not blinded to the timing of when to receive the intervention<br><br>8 – no confidence intervals were reported	4c – it is not mentioned whether the people analysing the outcomes were blinded
Ferwerda (2017)	1, 2, 3, 6, 7, 9, 10, 11	4a – participants were not blinded because they either received standard care or additional treatment<br><br>4b – investigators were not blinded because they had contact to participants<br><br>5 – intervention group showed significantly less<br><br>negative mood, lower levels of self-care, mobility, and lower impact of RA on daily life<br><br>8 – no confidence intervals were reported	4c – it is not mentioned whether the people analysing the outcomes were blinded
Khan (2020)	1, 2, 3, 5, 6, 7, 10, 11	4a - randomisation groups were made known to participants<br><br>4b – investigators were not blinded because they had contact to participants<br><br>8 – no confidence intervals were reported<br><br>9 – no effect size was calculated	4c – it is not mentioned whether the people analysing the outcomes were blinded

Study	Positive/<br><br>Methodologically sound	Negative/Relatively poor methodology	Unknowns
Knudsen (2024)	1, 2, 3, 5, 6, 7, 8, 10, 11	4a – participants were not blinded, intervention group received digital-, and control group face-to-face patient education<br><br>4b – investigators were not blinded, intervention and control group were treated differently<br><br>9 – no effect size was calculated	4c – it is not mentioned whether the people analysing the outcomes were blinded
Kurt (2024)	1, 2, 4b, 5, 6, 7, 8, 9, 10, 11	4a – participants were not blinded, intervention group received additional counselling	3 – sample size calculations are not mentioned<br><br>4c – it is not mentioned whether the people analysing the outcomes were blinded
Li (2025)	1, 2, 3, 4b, 4c, 5, 6, 7, 8, 9, 10, 11	4a – participants were not blinded to the timing of when to receive the intervention	
Li (2020)	1, 2, 3, 4c, 5, 6, 7, 8, 9, 10, 11	4a – participants were not blinded to the timing of when to receive the intervention<br><br>4b – investigators were not blinded	
Lorig (2008)	1, 3, 5, 6, 7, 9, 10, 11	4a – participants were not blinded, intervention group received additional treatment<br><br>4b –investigators were not blinded<br><br>8 – no confidence intervals were reported	2 – it is mentioned that participants were randomised but not mentioned how the randomisation was carried out<br><br>4c – it is not mentioned whether the people analysing the outcomes were blinded
Pouls (2022)	1, 2, 3, 4c, 5,6, 7, 8, 10, 11	4a – participants were not blinded to the timing of when to receive the intervention<br><br>4b – investigators were not blinded<br><br>9 – no effect size was calculated	

Study	Positive/<br><br>Methodologically sound	Negative/Relatively poor methodology	Unknowns
Rodríguez Sánchez-Laulhé (2022)	1, 2, 3, 4b, 6, 7, 8, 9, 10, 11	4a – participants were not blinded, intervention group received additional treatment<br><br>5 – differences between groups in the pain and satisfaction domains of a questionnaire	4c – it is not mentioned whether the people analysing the outcomes were blinded
Shigaki (2013)	1, 3, 5, 6, 7, 9, 10, 11	4a – participants were not blinded to the timing of when to receive the intervention<br><br>8 – no confidence intervals were reported	2 – it is mentioned that participants were randomised but not mentioned how the randomisation was carried out<br><br>4b – it is not mentioned whether the investigators were blinded<br><br>4c – it is not mentioned whether the people analysing the outcomes were blinded
Song (2022)	1, 2, 3, 4b, 5, 6, 7, 8, 9, 10, 11	4a – participants were not blinded, intervention group received additional treatment	4c – it is not mentioned whether the people analysing the outcomes were blinded
van den Berg (2006)	1, 2, 3, 4b, 5, 6, 7, 8, 9, 10, 11	4a – participants were not blinded, intervention group received additional treatment	4c – it is not mentioned whether the people analysing the outcomes were blinded
Zuidema (2019)	1, 2, 3, 5, 6, 7, 8, 9, 10, 11	4a – participants were not blinded, intervention group received additional treatment<br><br>4b – investigators were not blinded, they informed participants of allocation to either control or intervention group	4c – it is not mentioned whether the people analysing the outcomes were blinded
