# Supplementary material for: Potential of digital applications for self-management and other outcomes in inflammatory rheumatic diseases: a systematic literature review
Source: Front Med (Lausanne). 2025 Jul 9;12:1617151. doi: 10.3389/fmed.2025.1617151 (PMC12285587; doi:10.3389/fmed.2025.1617151)
Supplement: Supplementary file 3 [file Table_3.pdf]

Supplementary Material

Supplementary File S3 Overview and outcomes of included studies

First author (year)	Participants (% female), mean (SD) age in years	Intervention, duration	Control intervention	Outcomes of interest	Results relevant to systematic review
Allam (2015)	Adults with RA from Switzerland, aged ≥18, n=155 (45.8), age 58 (12.3)	Website ONESELF: 4 groups with access to different sections and features of website<br>- 1st group: informational sections only<br>- 2nd group: informational- and social support sections<br>- 3rd group: informational- and gaming sections<br>- 4th group: informational-, social support- and gaming sections<br>Duration: 16 wks	No access to ONESELF	- Self-management and self-care (self-empowerment and physical activity)	Self-management (Self-empowerment)<br>- significant increase in 2nd and 4th group compared to control group over time<br>- 2nd: B=2.59, *p* = 0.03<br>- 3rd: B=2.29, p = 0.05<br>Physical activity<br>- mean minutes spent on exercise increased for 4th group (B=3.39, *p* = 0.02)
Allen (2021)	Adults with SLE from the USA of UNC Health Care system, aged ≥18, n=60 (95), age: 49[a] (n.r.), PTU n=15 (100%), age: 51 (14)	PainTRAINER: internet-based version of pain coping skills training<br>- guided feedback, interactive exercises, animated demonstrations, automated email reminders<br>- 8 modules (one per week, 30–45 min long)<br>Duration: 8 wks	Received intervention after completion of follow-up assessments	- Pain<br>- Depression and anxiety<br>- Functional impairment<br>- Fatigue<br>- HRQoL	(Only 50% of IG logged into program (pain trainer users (PTU), n=15))<br>Pain<br>- PROMIS:<br>- IG: mean change (SD): −2.6 (6.6), *d* = −0.12<br>- CG: mean change (SD): −1.7 (790)<br>- PTU: mean change (SD) −3.9 (6.5), *d* = −0.30<br>- Catastrophising:<br>- IG: mean change (SD): 2.3 (9.6), *d* = −0.16<br>- CG: mean change (SD): 3.6 (6.5)

First author (year)	Participants (% female), mean (SD) age in years	Intervention, duration	Control intervention	Outcomes of interest	Results relevant to systematic review
					- PTU: mean change (SD): −0.9 (8.9), *d* = −0.60<br>Depression and anxiety<br>- Depression:<br>- IG: mean change (SD): −3.4 (8.6), *d* = −0.32<br>- CG: mean change (SD): −0.6 (9.0)<br>- PTU: mean change (SD): −4.1 (5.2), *d* = −0.44<br>- Anxiety:<br>- IG: Mean change (SD) 1.4 (9.3), *d* = 0.09<br>- CG: Mean change (SD) 0.4 (11.2)<br>- PTU: Mean change (SD) −0.7 (8.1), *d* = −0.11<br>Functional impairment<br>- IG: mean change (SD): − 3.8 (4.1), *d* = − 0.56<br>- CG: mean change (SD): − 0.6 (7.0)<br>- PTU: mean change (SD): −4.0 (4.4), *d* = −0.55<br>Fatigue<br>- Fatigue:<br>- CG: mean change (SD) -3.5 (7.1)<br>- IG: mean change (SD) -2.2 (8.2), *d* = 0.17<br>- PTU: mean change (SD) -5.1 (6.2), *d* = -0.23<br>HRQoL (sleep, Ability to Participate in Social Roles and Activities, general)<br>- Sleep:<br>- CG: mean change (SD) 0.4 (9.3)<br>- IG: mean change (SD) 0.6 (9.4), *d* = 0.02<br>- PTU: mean change (SD) -2.1 (7.8), *d* = -0.28

First author (year)	Participants (% female), mean (SD) age in years	Intervention, duration	Control intervention	Outcomes of interest	Results relevant to systematic review
					- Ability to Participate in Social Roles and Activities:<br>- CG: mean change (SD) 0.5 (6.8)<br>- IG: mean change (SD) 0.7 (6.5), *d* = 0.03<br>- PTU: mean change (SD) 0.8 (7.4), *d* = 0.05<br>- General:<br>- IG: mean change (SD): 2.0 (13.3), ES = 0.04<br>- CG: mean change (SD) 1.4 (12.0)<br>- PTU: mean change (SD): 4.9 (11.1), *d* = 0.30
Ferweda (2017)	Adults with RA and heightened levels of psychological distress from the Netherlands, aged ≥18, n=133 (64), age: 56.4 (10)	Internet-based cognitive behavioural intervention on top of standard care<br>- 1-4 tailored intervention modules (pain and functional disability, fatigue, negative mood or social functioning)<br>- assignments, psychoeducational texts and cognitive strategies<br>Duration: between 9 and 65 wks (mean=26, SD 12)	Standard rheumatological care<br>- yearly check-ups + possibly 3 or 6 monthly check-ups<br>- physical therapy, occupational therapy, care by specialised rheumatology nurses	- Depression and anxiety<br>- Pain<br>- Functional impairment<br>- Self-care<br>- Disease activity<br>- Fatigue<br>- HRQoL	<u>Depression and anxiety</u><br>- Depression:<br>- Significant decrease in IG ($F(1, 97.25) = 19.48$, $p < 0.001$ compared to CG<br>- Anxiety:<br>- Significant decrease in IG: $F(1, 103.76) = 11.45$, $p < 0.001$ compared to CG<br><u>Pain</u><br>- No significant group differences ($p = 0.35$)<br><u>Functional impairment</u><br>- No significant group differences ($p = 0.17$)<br><u>Self-care</u><br>- No significant group differences ($p = 0.19$)<br><u>Disease activity</u><br>- No significant group differences ($p = 0.79$)<br><u>Fatigue</u><br>- No significant group differences (p = 0.24)<br><u>HRQoL</u>

First author (year)	Participants (% female), mean (SD) age in years	Intervention, duration	Control intervention	Outcomes of interest	Results relevant to systematic review
					- decrease in IG compared to CG: *F*(1, 104.19) = 2.91, $p = 0.09$, *d* =0.18
Khan (2020)	Adults with SLE from the USA taking a stable dose of 1 or more rheumatological drugs for 3 or more months prior to enrolment, aged ≥18, n=46 (96), age: 43 (n.r.)	Smartphone app<br>- tracking lifestyle activities, symptoms<br>- software that analyses and organises data<br>- web portal that presents data to health coach<br>Duration: 16 wks	Standard rheumatological care	- Fatigue<br>- Pain<br>- Functional impairment	(results of people who completed 10 or more sessions, submitted end-of-study data and experienced no exclusions)<br>**ITT analyses**<br><u>Fatigue (FACIT)</u><br>- No significant between-group difference: 4.5, $p = 0.17$<br><u>LupusQoL-Fatigue</u><br>- No significant between-group difference (9.4, $p = 0.22$)<br><u>Pain (Severity)</u><br>- No significant between-group difference: 4.5 ($p = 0.17$)<br><u>Pain interference</u><br>- No significant between-group difference; -0.7, $p = 0.31$<br><u>Functional impairment</u><br>- No significant between-group difference, -3.1, $p = 0.99$<br><br>**PP analyses**<br><u>Fatigue (FACIT)</u><br>- Significant between-group difference: 18.0, $p < 0.001$<br>- Decrease in IG (Baseline: 20.5 (10.3, 26.8), End of Programme (EOP): 43.5 (28.5, 47.8), change: +17.5, $p = 0.001$) compared to CG (Baseline: 20.5 (14.0, 27.3), EOP: 22.0 (12.5, 28.3), change: -0.5, $p = 0.79$)<br><u>LupusQoL-Fatigue</u><br>- Significant between-group difference (25.0, $p = <0.001$)

First author (year)	Participants (% female), mean (SD) age in years	Intervention, duration	Control intervention	Outcomes of interest	Results relevant to systematic review
					- Improvement in IG (Baseline 28.1 (25.0, 53.1), EOP: 81.3 (64.1, 92.2), change: 37.5 (21.9, 48.4) compared to CG (Baseline 25.0 (10.9, 43.8), EOP: 34.4 (25.0, 53.1), change: 12.5 (-1.6, 20.3)<br>Pain severity<br>- Significant between-group difference: -1.9, $p = 0.049$<br>- Decrease in IG (Baseline: 4.8 (3.0, 6.5), EOP: 3.3 (1.8, 5.2), Change: −1.3, $p = 0.02$), compared to CG (Baseline: 3.9 (2.9, 5.9), EOP: 3.8 (2.6, 6.4), change: 0.6, $p = 0.68$)<br>Pain interference<br>- Significant between-group difference: -2.5, $p = 0.02$<br>- Reduction in IG (Baseline: 6.3 (4.0, 7.5), EOP: 2.0 (0.5, 5.3), Change: −2.5, $p = 0.003$), compared to CG (Baseline: 5.4 (4.1, 6.3), EOP: 4.9 (1.6, 6.5), change: 0, $p = 0.64$)<br>Functional impairment<br>- Significant between-group difference: 14.1, p = 0.49<br>- Increase in IG (Baseline: 51.6 (34.4, 61.7), EOP 71.9 (37.5, 93.0), change: 17.2, $p = 0.02$, compared to CG (Baseline: 51.6 (27.3, 65.6), EOP: 48.4 (26.6, 71.1), change: 3.1, $p = 0.66$
Knudsen (2024)	Patients with RA diagnosis ≤3 months from Denmark, aged ≥18, n=175 (61), age: 59 (n.r.)	Digital patient education programme regarding disease-specific knowledge<br>- Mandatory module: typical disease course, causes, symptoms, treatment	Standard rheumatological care<br>- 1-hour session of face-to-face patient education by rheumatology nurse	- Self-management and self-care (Self-efficacy)<br>- Medication adherence<br>- HRQoL<br>- Disease activity	Self-efficacy<br>- Significant average difference in outcomes between groups from baseline to 12 month<br>- IG: 4.34-point greater improvement 95%-CI[-8.17, -0.51], $p = 0.026$)

First author (year)	Participants (% female), mean (SD) age in years	Intervention, duration	Control intervention	Outcomes of interest	Results relevant to systematic review
		- optional modules: medical treatment, potential comorbidities, physical and radiological examinations, guidance and inspiration for managing symptoms, coping with RA in everyday life<br>Duration: 1 year			Medication adherence<br>- no significant differences between groups from baseline to 12 months in odds for low adherence (p = 0.501)<br>HRQoL<br>- No significant between-group differences<br>Disease activity at 12 months<br>- IG: median (IQR): 2.0 (1.6-3.0)<br>- CG: median (IQR): 2.4 (1.8-3.1)
Kurt (2024)	Patients with RA, SpA, PsA from Germany, aged ≥18, n=158 (72.8), age: 53.3 (11.7)	Mida Rheuma App (individual lifestyle counselling)<br>- Assess patient related outcomes<br>- individualised lifestyle counselling<br>- monitor disease burden<br>- healthy mediterranean diet<br>- sports and physical activity<br>- mental health<br>- non-smoking<br>- action plans 7-11 days long<br>Duration: 12 wks	Usage of Mida Rheuma app to assess patient related outcome measures (PROMs) only	- Disease activity<br>- Self-management (physical activity)<br>- Depression<br>- Functional impairment<br>- Pain	Disease activity<br>- Significant increase in odds ratio of achieving low disease activity or remission for IG: OR = 2.8, 95%-CI[1.1, –7.2], p = 0.035)<br>- no significant effect in CG (OR = 2.1, 95%-CI[0.9, –5.0], p = 0.097)<br>Physical activity<br>- No significant group differences in weekly time spent for physical activity (IG: β = 0.03, (−0.28, 0.34), p = 0.85; CG: β = −0.09, 95%-CI[−0.38, 0.2], p = 0.54) or in exercise questionnaire scores (IG: β = −13.1, 95%-CI[−61.2, 34.8], p = 0.59; CG: β = −6.3, 95%-CI[−51.6, 39.3], p = 0.79)<br>Depression<br>- No significant group differences (IG: β = 0.23, 95%-CI[−0.25, 0.71], p = 0.34, CG: β = 0.23, 95%-CI[−0.22, 0.69], p = 0.32)<br>Physical function<br>- no significant changes over time in the 2 groups (IG: β = 0.7, 95%-CI[−2.4, 3.9], p = 0.6; CG: β = −0.2, 95%-CI[−3.2, 2.7], p = 0.9)

First author (year)	Participants (% female), mean (SD) age in years	Intervention, duration	Control intervention	Outcomes of interest	Results relevant to systematic review
					<u>Pain</u><br>- no significant changes over time in groups IG: β = 3.4, 95%CI [−1.2, 8.0], *p* = 0.2; CG: β = −0.6, 95%-CI [−4.9, 3.8], *p* = 0.8)
Li (2025)	Patients with RA from Canada, aged ≥18, n=131 (91.6), age: 55.8 (13.1)	Monitoring app OPERAS<br>- monitor disease activity, symptoms, medication use, fatigues, depression, sleep quality<br>- activity tracking by pairing with a Fitbit (wearable device)<br>- 6 physical therapist counselling sessions over phone<br>- 2-h physical activity session with group and individual counselling<br>Duration: 26 wks	monthly e-newsletter unrelated to RA, standard medical treatment, received intervention after 27 wks (waitlist)	- Self-management<br>- Self-management (physical activity)<br>- Disease activity<br>- Pain<br>- Fatigue<br>- Depression	<u>Self-management</u><br>- IG: scores improved from baseline (65.1 (*SD* = 13.7)), to 27 wks (69.8 (*SD* = 17.2)), decreased at 53 wks (67.3 (*SD* = 15.1))<br>- CG: scores remained stable from baseline (68.3 (SD=13.9)), to 27 wks (67.1 (*SD* = 13.5)), to 53 wks (72.2 (*SD* = 17.5)<br>- Significant improvement in IG compared to CG at 27 weeks, (mean difference=6.2, 95%-CI[1.3, 11.1], *p < 0.05*))<br>- Intervention effect observed with adjusted coefficient of 5.3, 95% CI [2.0, 8.7], *p*≤0.0001<br><u>Self-management (physical activity)</u><br>- No statistically significant effects<br><u>Disease activity</u><br>- Intervention effect observed with an adjusted coefficient of -0.6, 95%-CI[-1.1, -0.2], *p* = 0.005)<br><u>Pain</u><br>- No statistically significant effects<br><u>Fatigue</u><br>- Intervention effect observed with an adjusted coefficient of -0.3, 95%-CI[-0.5, -0.1], *p* < 0.01<br><u>Depression</u><br>- IG: score improved from baseline (7.6 (SD = 5.6)) to 27 weeks (5.0 (SD = 3.8)) to 53 weeks (4.8 (SD = 4.0))

First author (year)	Participants (% female), mean (SD) age in years	Intervention, duration	Control intervention	Outcomes of interest	Results relevant to systematic review
					- CG: baseline (7.0 (SD = 4.9)), 27 wks (6.3 (SD = 5.3)), 53 wks (5.4 (SD = 5.0))<br>- Significant between-group difference at 27 wks compared to baseline −1.8, 95%-CI[−3.3, −0.2], p > 0.05<br>- Intervention effect observed with an adjusted coefficient of -1.3, 95%-CI[-2.3, -0.3], *p* = 0.01
Li (2020)	Patients with RA or SLE from Canada, aged ≥18, n=118 (91.6), age: 55.8 (13.1)	- in-person session with 20 minutes group education, 30 minutes individual counselling with physiotherapist (PT)<br>- use of Fitbit with account access<br>- 4 bi-weekly phone calls (20-30 mins) from PT<br>- app that showed physical activity goal attainment<br>Duration: 8 wks	Received intervention after 10 wks (waitlist)	- Self-management (physical activity)<br>- Pain<br>- Fatigue<br>- Depression	<u>Self-management (physical activity)</u><br>- IG: mean minutes per day in activity increased from baseline (37.8) to 9 wks (44.7), decreased to 18 wks (43.2) and to 27 wks (37.8)<br>- CG: mean minutes per day in activity at baseline (31.6), 9 wks (31.6), 18 wks (32.8), 27 wks (34.0)<br>- between-group adjusted difference was not significant (9.4 mins/day), 95%-CI[-0.5, 19.3], *p* = 0.06<br><u>Pain</u><br>- Statistically significant adjusted mean difference: -2.45, 95%-CI[-4.78, -0.13]) for IG compared to CG, *p* = 0.04<br><u>Fatigue</u><br>- No significant group differences<br><u>Depression</u><br>- IG: modest improvement from baseline: mean (SD) 7.6 (5.9) to 9 wks: mean (SD) 6.8 (5.6) and 27 wks: mean (SD) 5.2 (3.8)<br>- CG: modest improvements from baseline: mean (SD) 8.1 (5.6) to 9

First author (year)	Participants (% female), mean (SD) age in years	Intervention, duration	Control intervention	Outcomes of interest	Results relevant to systematic review
					wks: mean 7.9 (5.7) to 27 wks 6.7 (5.9)<br>- No significant between-group difference from baseline to 9 wks: −0.22, 95%-CI[−1.78, 2.35], p > 0.05
Lorig (2008)	Patients with RA from Canada, aged ≥18, n=144 (n.r.), age: n.r. (n.r.)	Internet-based arthritis self-management programme<br>- Learning center<br>- Discussion Center<br>- exercise logs<br>- medication diaries<br>- exercise programmes<br>- Arthritis Helpbook<br>Duration: 6 wks	Standard care and $10 Amazon.com gift card for each questionnaire completed	- Pain<br>- Self-management (Self-efficacy)<br>- Self-management (physical activity)<br>- Functional impairment<br>- Work productivity impairment<br>- Fatigue	(Only one-year changes for IG and CG in RA patients were considered)<br><u>Pain</u><br>- significant difference in IG: mean (SD) −0.514 (2.79) compared to CG: mean (SD) −0.069 (1.69), *p* = 0.040<br><u>Self-efficacy</u><br>- no significant increase in IG: mean (SD) 0.783 (1.32) compared to CG: mean (SD) 0.242 (1.59), *p* = 0.282<br><u>Physical activity</u><br>- Aerobic Exercise (minutes per week): no significant difference between IG: mean (SD) −10.1 (98.0) and CG: mean (SD) −1.18 (98.8), *p* = 0.963<br>- Stretching and Strength Exercise (minutes per week): no significant difference between IG: mean (SD) 3.99 (54.7) and CG: mean (SD) −1.67 (54.2), *p* = 0.877<br><u>Functional impairment</u><br>- No significant difference (*p* = 0.850), between IG mean change (SD): -0.003 (0.406) and CG mean change (SD): 0.024 (0.332) in disability score<br><u>Work productivity impairment</u><br>- significant difference (*p* = 0.003), between IG mean change (SD): -0.366 (1.02) and CG mean

First author (year)	Participants (% female), mean (SD) age in years	Intervention, duration	Control intervention	Outcomes of interest	Results relevant to systematic review
					change (SD): 0.132 (0.734) in activity limitation score<br>Fatigue<br>- No significant difference (*p* = 0.925), between IG mean change (SD): -0.361 (2.00) and CG mean change (SD): 0.056 (1.94)
Pouls (2022)	Patients with RA (using disease modifying anti-rheumatic drugs) from the Netherlands aged ≥18, n=221 (73), age: 61 (12)	Game-based intervention<br>- crossword<br>- sudoku<br>- word search<br>- Tangram<br>- to unlock games or puzzles, players completed behavioural tasks<br>Duration: 12 wks	Standard care<br>- regular consultations with rheumatologist<br>- received intervention after 12 wks (waitlist)	- Disease activity<br>- Functional impairment<br>- Medication adherence	Disease activity<br>- no significant difference between IG: median 2.5, IQR (1.5–4.2) and CG: median 2.5 IQR (1.2–4.0), group difference: 0.0, 95%-CI[−0.8, 0.8]<br>Functional impairment<br>- no significant difference between IG: median 0.6, IQR (0.3–1.4) and CG: median 0.8, IQR (0.3–1.4), group difference: −0.1, 95%-CI[−0.5, 0.2]<br>Medication adherence<br>- no significant difference between IG: mean (SD) 73 (11) and CG: mean (SD) 75 (12), group difference: 2.2, 95%-CI[−1.1, 5.5]
Rodríguez Sánchez-Laulhé (2022)	Patients with RA (of hands, wrists, or fingers, having RA ≥2 years, report current pain and disability) from Spain, aged ≥18, n=30 (61), age: 55.8 (13.1)	App "CareHand"<br>- exercise videos<br>- exercise diary<br>- advice on diet and joint protection, self-management<br>- 4 times a week, 15-20 minutes<br>- telephone follow-up calls<br>Duration: 12 wks	Standard care<br>- written exercise programme and recommendations	- Pain<br>- Functional impairment<br>- Work productivity impairment	Pain<br>- At baseline differences between groups ($p = 0.001$)<br>- significant time x group effect: $F(2, 106)=13.918$, $p < 0.001$; $\eta^2 = 0.21$)<br>- no significant change over time in pain intensity $F(3, 153) = 1.352$; $p = 0.26$, $\eta^2 = 0.03$<br>Functional impairment (hand function)<br>- significant time×group effect $F(1.62, 85.67)=9.163$; $p < 0.001$; $\eta^2 = 0.15$)

First author (year)	Participants (% female), mean (SD) age in years	Intervention, duration	Control intervention	Outcomes of interest	Results relevant to systematic review
					- mean differences between groups at third-month follow-up: 16.86 points, 95%-CI[8.70, -25.03], p =0.001<br>- mean differences between groups at six-month follow-up: 17.21 points, 95%-CI[4.78, -29.63], p =0.007<br>Work productivity impairment<br>- significant between-group differences<br>- 3 months difference: 10.97, 95%-CI[-4.70, 26.64], p = 0.17<br>- 6 months difference: 23.83, 95%-CI[5.77, 41.90], p = 0.01
Shigaki (2013)	Patients with RA (with stable RA medication regimen for 3 months) from the USA aged ≥18, n=106 (93), age: 50 (n.r.)	Website RAHelp<br>- self-management and educational programme<br>- social networking applications (news feature)<br>- assessment tools<br>- monitoring tools (to-do list)<br>- database for leaders<br>- weekly telephone support for 15-30 minutes<br>Duration: 10 wks	Received intervention after 10 weeks (waitlist)	- Functional impairment<br>- Self-management (self-efficacy)<br>- Depression<br>- QoL<br>- Pain	Functional impairment<br>- Post-intervention: no significant difference between IG: mean (SD) 1.7 (1.6) and CG: mean (SD) 1.9 (1.7), ES = 0.56, p = 0.065<br>- 9-Month Follow-Up: no significant difference between IG: mean (SD) 1.6 (1.6) and CG: mean (SD) 1.6 (1.5), ES = 0.48, p = 0.16<br>Self-Efficacy<br>- Post-intervention: significant improvement in IG: mean (SD) 83.9 (19.0) compared to CG: mean (SD) 68.5 (23.8), ES = 0.92, p < 0.01<br>- 9-Month Follow-Up: significant improvement in IG: mean (SD) 84.1 (16.3) compared to CG: mean (SD) 68.6 (23.3), ES = 0.92, p < 0.01<br>Depression<br>- Post-Intervention: no significant improvement in IG: mean (SD)

First author (year)	Participants (% female), mean (SD) age in years	Intervention, duration	Control intervention	Outcomes of interest	Results relevant to systematic review
					9.8 (7.6) compared to CG: mean (SD) 11.9 (11.2), *ES* = 0.44, *p* = 0.14<br>- 9-Month Follow-Up: no significant improvement in IG: mean (SD) 10.8 (8.2) compared to CG: mean (SD) 13.2 (11.2), *ES* = 0.49, *p* = 0.14<br><u>QoL</u><br>- Post-Intervention: significant improvement in IG: mean (SD) 88.4 (11.7) compared to CG: mean (SD) 84.9 (14.6), *ES* = 0.66, *p* = 0.003<br>- 9-Month Follow-Up: significant improvement in IG: mean (SD) 88.0 (11.8) compared to CG: mean (SD) 83.1 (16.0), *ES* = 0.71, *p* = 0.004<br><u>Pain</u><br>- 4-Week Pain (4WP), Post-Intervention: no significant improvement in IG: mean (SD) 4.2 (2.1) compared to CG: mean (SD) 4.7 (2.5), *ES* = 0.57, *p* = 0.07<br>- 4WP 9-Month Follow-Up: no significant improvement in IG: mean (SD) 4.1 (2.6) compared to CG: mean (SD) 4.3 (2.5), *ES* = 0.31, *p* = 0.34<br>- Current Pain Today (CPT)- Post-Intervention: no significant improvement in IG: mean (SD) 36.8 (28.3) compared to CG: mean (SD) 40.2 (31.2), *ES* = 0.37, *p* = 0.24<br>- CPT- 9-Month Follow-Up: no significant improvement in IG:

First author (year)	Participants (% female), mean (SD) age in years	Intervention, duration	Control intervention	Outcomes of interest	Results relevant to systematic review
					mean (SD) 41.4 (31.2) compared to CG: mean (SD) 39.2 (29.6), *ES* = 0.19, *p* = 0.58
Song (2022)b	Patients with AS from China, aged ≥14, n=118 (21.2%), age: 29.9 (8.23)	Social networking app "WeChat"<br>- Educational information regarding knowledge of AS, medication, exercise, daily life management, psychological support, self-assessment<br>- 4 individual educational sessions via WeChat<br>Duration: 12 wks	Standard care<br>- basic health advice via paper handouts and in person by a nurse	- Self-management (self-efficacy)<br>- Disease activity<br>- Functional impairment	<u>Self-efficacy</u><br>- Posttest: significant improvement in IG: mean (SD) 7.60 (1.5) compared to CG: mean (SD) 6.41 (2.04), t=3.612, *p* < 0.001<br><u>Disease activity</u><br>- posttest: no significant improvement in IG: mean (SD) 2.95 (1.71) compared to CG: mean (SD) 3.41 (1.76), t= -1.434, *p* = 0.15<br><u>Functional impairment</u><br>- posttest: no significant improvement in IG: median 1.00 (IQR = 1.40) compared to CG: median 1.40 (IQR = 1.60), *U* = –1.764, *p* = 0.08
van den Berg (2006)	Patients with RA from the Netherlands, aged ≥18, n=160 (76.5), age: 49.6[a] (n.r.)	Website "cybertraining.nl"<br>- physical activity programme<br>- Individualised training (IT) group received: personalised exercises *(*muscle strengthening, range-of-motion, cycling), bicycle ergometer, advice for additional physical activities, quarterly group meetings, individual supervision by physical therapists<br>Duration: 1 year	General training (GT) group received general exercises (aerobic, muscle strengthening, range-of-motion), physical activity advice, informational CD-ROM	- Self-management (physical activity)<br>- HRQoL<br>- Disease activity<br>- Functional impairment	<u>Physical activity</u><br>- Moderate activity 30 mins, ≥5 days/wk: significantly higher proportion of people from IG (38% vs. 22% at 6 months, *p* = 0.041 and 9 months (35% vs. 11%), *p* < 0.001)<br>- Vigorous activity 20 mins, ≥3 days/wk: significantly higher proportion of people from IG at 6 months (35% vs. 13%), 9 months (40% vs. 14%), and 12 months (34% vs. 10%), *p* < 0.001<br><u>HRQoL</u><br>- No significant difference between groups in changes over one year: *p* = 0.12<br>- 12 months, IG improved by median of -1.3 (IQR:-2.35, -0.34),

First author (year)	Participants (% female), mean (SD) age in years	Intervention, duration	Control intervention	Outcomes of interest	Results relevant to systematic review
					and CG by -0.6 (IQR: -1.37, -0.24)<br>Disease activity<br>- No significant difference between groups in change over one year; *p* = 0.63<br>- 12 months, IG improved by median of -0.4 (IQR: -0.6, -0.1) and CG by -0.5 (IQR: -0.7, -0.2)<br>Functional impairment<br>- No significant difference between groups in change over one year; *p* =0.41<br>- 12 months, IG improved by median of -0.09 (IQR: -0.16, -0.01) and CG by -0.04 (-0.11, 0.04)
Zuidema (2019)	Patients with RA from the Netherlands, aged ≥18, n=157 (n.r.) age: 62[a] (n.r.)	Web-based programme<br>- modules with videos, information, exercises (Balancing activity and rest, setting boundaries, asking for help and social support, use of medicines, communication with health professionals, use of assistive devices, performing physical exercises, coping with worries, coping with RA)<br>- diary to track symptoms<br>Duration: 1 year	Standard care	- Self-management<br>- Functional impairment<br>- Pain<br>- Fatigue	Self-management<br>- Patient activation (PAM-13): no significant between-group difference at 6-months: -0.7, 95%-CI[-3.4, 1.5], *p* = 0.44, *d* = 0.00, as well as at 12-months: -0.1, 95%-CI[-1.6, 1.5], *p* = 0.93, *d* = 0.00<br>- Self-management ability (SMAS-S): no statistical between-group difference at 6-months: 0.3, 95%-CI[-1.4, 2.0], *p* = 0.72, *d* = 0.00, as well at 12-months: 0.7, 95%-CI[-1.1, 2.5], *p* = 0.43, *d* = 0.03<br>- self-efficacy: non-significant changes over time in both groups<br>Functional impairment<br>- No significant between-group difference at 6-months: 2.5, 95%-CI[-3.3, 8.1], *p* = 0.40, *d* = 0.00

First author (year)	Participants (% female), mean (SD) age in years	Intervention, duration	Control intervention	Outcomes of interest	Results relevant to systematic review
					and 12-months: -0.2, 95%-CI[-5.4, 5.1], *p* = 0.96, *d* = 0.00<br>Pain<br>- no significant between-group difference in pain today after 6-months:0.0, 95%-CI[-0.6, 0.7], *p* = 0.97, *d* = 0.00 and 12 months: 0.5, 95%-CI[-0.1, 1.2], *p* = 0.13, *d* = 0.10<br>- no significant between-group difference in pain last 2 wks after 6 months (*p* = 0.97, *d* = 0.00) and 12 months (*p* = 0.60, *d* = 1.13)<br>Fatigue<br>- no statistical between-group difference in fatigue today (NRS) at 6-months: 0.2, 95%-CI[-0.5, 0.8], *p* = 0.66, *d* = 0.00, and at 12-months: 0.03, 95%-CI[-0.4, 0.9], *p* = 0.46, *d* = 0.01<br>- no statistical between-group difference in mean fatigue in last 2 weeks (NRS) at 6-months: -0.23, 95%-CI[-0.9, 0.4], *p* =0.45, *d* =-0.1 and 12 months: 0.1, 95%-CI [-0.6, 0.7], *p* =0.81, *d* =0.0<br>- no statistical between-group difference in MCPI-F at 6-months: 0.1, 95%-CI[-0.8, 0.9], *p* =0.90, *d* =0.00 and at 12 months: 0.3, 95%-CI[-0.7, 1.2], *p* =0.58, *d* =0.01

[a] Value is weighted using a formula, as only the mean age and standard deviation were provided per group, not for the overall sample
[b] Study was included because reported mean ages and SD of total sample was: 29.9 (SD 8.23), intervention group: 30.8 (SD 8.82) and control group: 29.1 (SD 7.58), demonstrated that majority of participants were significantly older than 14

AS, ankylosing spondylitis; B, unstandardized beta coefficient; β, regression coefficient; CG ,control group; CI, confidence interval; *d*, Cohen's effect size; EOP, end of programme; ES, effect size; ANOVA, analysis of variance, HR-QoL, health-related quality-of-life; IG, intervention group; IQR, interquartile range; MPCI-F, Modified Pain Coping Inventory for Fatigue; n, total number; η^2, measure of effect size; n.r., not reported, NRS, numerical rating scale; PA, physical activity; PsA psoriatic arthritis; PT, physical therapist; PTU, PainTRAINER users; QoL,quality of life; RA, rheumatoid arthritis; SD, standard deviation, SLE, systemic lupus erythematosus; SpA, spondyloarthritis; t, independent sample t test; U, Mann-Whitney U test; UNC, University of North Carolina at Chapel Hill; wk, week
