# Supplementary material for: Potential of digital applications for self-management and other outcomes in inflammatory rheumatic diseases: a systematic literature review
Source: Front Med (Lausanne). 2025 Jul 9;12:1617151. doi: 10.3389/fmed.2025.1617151 (PMC12285587; doi:10.3389/fmed.2025.1617151)
Supplement: Supplementary file 4 [file Table_4.pdf]

Supplementary Material

Supplementary File S4 Results of digital interventions on secondary outcomes in IRD patients. Blue shading indicates a statistically significant improvement (p ≤0.05) compared with the control group in at least one subgroup or an effect size (Cohen's *d*) ≥0.3 for studies that did not present p values.

First author (pub year)	Intervention	Duration	Assessment	Results
Disease activity				
Ferwerda (2017)	Internet-based cognitive behavioral intervention on top of standard care	9 to 65 wks (mean [SD], 26 [12] wks)	RADAI	No significant between-group difference in change over 12 months in ITT (p=0.79) or PP (p-value NR) analyses
Knudsen (2024)	Web-based digital patient education program regarding disease-specific knowledge	1 year	Disease Activity Score-28 joints	Slightly less disease activity at 12 months for IG vs control. Not all patients contributed data so a statistical test for difference between groups was not performed.<br><br>Median (IQR):<br>IG 2.0 (1.6, 3.0)<br>Control 2.4 (1.8, 3.1)
Kurt (2024)	Mobile app (Mida Rheuma) with individualized lifestyle counselling	12 wks	RADAI for patients with RA<br>DAPSA for PsA<br>BASDAI for SpA	Significant effect on the odds of achieving low disease activity or remission at week 12 in IG but not in control<br><br>OR (95% CI)<br>IG=2.8 (1.1, 7.2); p=0.035<br>Control=2.1 (0.9, 5.0); p=0.097
Li (2025)	Web/mobile app OPERAS and fitness tracker (Fitbit)	26 wks	RADAI	Significant difference between IG and control at 27 wks in GLMM but not in unadjusted analyses<br><br>Unadjusted: p >0.05 (exact p-value NR)<br>GLMM intervention effect: -0.6 (-1.1, 0.2), *d*=0.36, p=0.005

First author (pub year)	Intervention	Duration	Assessment	Results
Pouls (2022)	Mobile game-based intervention	12 wks	RADAI	No significant between-group difference at 12 wks (p-value NR)
Song (2022)	Mobile social networking app “WeChat”	12 wks	BASDAI	No significant between-group difference at 12 wks (p=0.15)
van den Berg (2006)	Website “cybertraining.nl” individualized physical activity program	1 year	Disease Activity Score-28 joints	No significant between-group difference over 12 months (p=0.63)
Work outcomes				
Lorig (2008)	Internet-based arthritis self-management program	6 wks	Activities Limitation Scale	Significant improvement in activity limitation at 1 year for IG vs control<br>Mean (SD) difference<br>IG: -0.366 (1.02)<br>Control: 0.132 (0.734)<br>p=0.003
Rodríguez Sánchez-Laulhé (2022)	Mobile CareHand app	12 wks	Michigan Hand Outcome Questionnaire work performance subscale	Significant improvement for IG vs control at 6 months (but not at 3 months; p=0.17)<br><br>Mean (95% CI) between-group difference at 6 months: 23.83 (5.77, 41.90), p=0.01<br>Significant time-group effect (p=0.006)
Medication adherence				
Knudsen (2024)	Web-based digital patient education program regarding disease-specific knowledge	1 year	Compliance Questionnaire for Rheumatology	No significant between-group difference in relative odds for low adherence from BL to month 12 for unadjusted (p=0.416) or adjusted[a] (p=0.501) analyses
Pouls (2022)	Mobile game-based intervention	12 wks	Compliance Questionnaire for Rheumatology	No significant between-group differences in rates of adherence at 1 month (p=0.06) or 3 months (p=0.13)
QoL				
Allen (2021)	Internet-based training on pain coping skills (PainTRAINER)<br><br>Only 50% of IG (15/30) logged into program (PainTRAINER users [PTU])	6 wks	PROMIS-29 sleep disturbance and participation subscales	Small improvement from BL to 9 wks in sleep disturbance in PTU group (*d*=-0.28), but not total IG (*d*=0.02)<br><br>No effect on ability to participate in social roles and activities in either group (*d*=0.03 for total IG and *d*=0.05 for PTU)

First author (pub year)	Intervention	Duration	Assessment	Results
			LupusPRO HR-QoL and non-HR-QoL scores	Moderate improvement from BL to 9 months in HR-QoL score in PTU group (*d*=0.30), but not total IG (*d*=0.04)<br><br>No effect on non-HR-QoL in either group (*d*=0.02 for total IG and *d*=-0.04 for PTU)
Ferwerda (2017)	Internet-based cognitive behavioral intervention	9 to 65 wks (mean [SD], 26 [12] wks)	Composite "impact on daily life" measure based on IRGL self-care and mobility scales and SF-36 physical/ emotional health problems scales	No significant between-group difference for IG vs control in ITT analysis of change over 12 months (*d*=0.18, P=0.09)<br><br>Significant difference between IG and control in PP analysis (P=0.049; *d* NR)
Knudsen (2024)	Web-based digital patient education program regarding disease-specific knowledge	1 year	EuroQol (EQ-5D-5L and VAS)	No significant between-group differences at 1 year in EQ-5D-5L (p=0.661) or VAS (p=0.672)
Shigaki (2013)	Website RAHelp	10 wks	Quality of Life Scale	Significant improvements for IG vs control immediately post-intervention and at 9-month follow-up[b]<br><br>Mean (SD) postintervention:<br>IG: 88.4 (11.7)<br>Control: 84.9 (14.6)<br>ES=0.66, p=0.003<br><br>Mean (SD) 9-month follow-up<br>IG: 88.0 (11.8)<br>Control: 83.1 (16.0)<br>ES=0.71, p=0.004
van den Berg (2006)	Website “cybertraining.nl” individualized physical activity program	1 year	Rheumatoid Arthritis Qual-ity of Life	No significant between-group differences over 12 months (p=0.12)

First author (pub year)	Intervention	Duration	Assessment	Results
Fatigue				
Allen (2021)	Internet-based training on pain coping skills (PainTRAINER)<br><br>Only 50% of IG (15/30) logged into program (PainTRAINER users [PTU])	6 wks	PROMIS Fatigue domain	Small improvement in change from BL to 9 wks in PTU group vs control (*d*=-0.23), but not for total IG (*d*=0.17)
Ferwerda (2017)	Internet-based cognitive behavioral intervention	9 to 65 wks (mean [SD], 26 [12] wks)	Checklist Individual Strength fatigue scale	No significant between-group difference in change over 12 months in ITT (*d*=0.24; p=0.06) or PP (p-value NR) analyses
Khan (2020)	Mobile smartphone app	16 wks	FACIT Fatigue domain	Significant improvement at 16 wks for IG in PP analysis, but not in ITT (p=0.17)<br><br>PP between-group difference: 18.0 (p<0.001)
			LupusQoL-Fatigue	Significant improvement at 16 wks for IG in PP analysis, but not in ITT (p=0.22)<br><br>PP between-group difference: 25.0 (p<0.001)
Li (2025)	Web/mobile app OPERAS and fitness tracker (Fitbit)	26 wks	Fatigue Severity Scale	Significant improvements for IG vs control in unadjusted and GLMM analyses at week 27<br><br>Unadjusted mean (95% CI) difference: -0.3 (-0.7, 0.0), p<0.05 (exact p-value NR)<br><br>GLMM intervention effect: -0.3 (-0.5, -0.1), *d*=0.32, p=0.01
Li (2020)	Fitness tracker (Fitbit) and web-based app FitViz	8 wks	Fatigue Severity Scale	No significant between-group differences at 9 wks (p>0.05) (exact p-value NR)
Lorig (2008)	Internet-based arthritis self-management program	6 wks	Numeric rating scales (self-designed)	No significant between-group differences for change from BL to one year (p=0.925)
Zuidema (2019)	Web-based self-management program	1 year	Modified Pain Coping Inventory for Fatigue	No significant between-group differences at 6 months (p=0.90) or 12 months (p=0.58)
			Numeric rating scale for fatigue today and mean fatigue in last 2 wks	No significant between-group differences for fatigue today (p=0.66) or mean fatigue in last 2 wks (p=0.45) at 6 months or 12 months (p=0.46 and 0.81)

[a]Adjusted for baseline age, sex, educational level, Disease Activity Score-28 joints, and site

[b]Effect size coefficients not reported

BASDAI, Bath Ankylosing Spondylitis Disease Activity Index; DAPSA, Disease Activity Psoriatic Arthritis; ES, effect size; FACIT, Functional Assessment of Chronic Illness Therapy; GLMM, Generalized Linear Mixed-effect Model; IG, interventional group; IRGL, Impact of Rheumatic Diseases on General Lifestyle; ITT, intention-to-treat; NR, not reported; OT, occupational therapy; PROMIS, Patient-reported Outcome Measurement Information System; PP, per protocol; PsA, psoriatic arthritis; PT, physical therapy; RA, rheumatoid arthritis; RADAI, Rheumatoid Arthritis Disease Activity Index; SpA, spondyloarthritis; SF, Short Form; VAS, visual analog scale; wk, week
